# Adhesion of Enteropathogenic, Enterotoxigenic, and Commensal Escherichia coli to the Major Zymogen Granule Membrane Glycoprotein 2

**DOI:** 10.1128/aem.02279-21

**Published:** 2022-03-08

**Authors:** Christin Bartlitz, Rafał Kolenda, Jarosław Chilimoniuk, Krzysztof Grzymajło, Stefan Rödiger, Rolf Bauerfeind, Aamir Ali, Veronika Tchesnokova, Dirk Roggenbuck, Peter Schierack

**Affiliations:** a Institute of Biotechnology, Faculty Environment and Natural Sciences, Brandenburg University of Technology Cottbus-Senftenberg, Senftenberg, Germany; b Department of Biochemistry and Molecular Biology, Faculty of Veterinary Medicine, Wrocław University of Environmental and Life Sciences, Wrocław, Poland; c Department of Genomics, Faculty of Biotechnology, University of Wrocław, Wrocław, Poland; d Institute for Hygiene and Infectious Diseases of Animals, Justus Liebig University, Giessen, Germany; e National Institute for Biotechnology and Genetic Engineering College, Pakistan Institute of Engineering and Applied Sciences (NIBGE-C, PIEAS), Faisalabad, Pakistan; f Department of Microbiology, University of Washington, Seattle, Washington, USA; g Faculty of Health Sciences Brandenburg, Brandenburg University of Technology Cottbus-Senftenberg, Senftenberg, Germany; University of Illinois at Urbana-Champaign

**Keywords:** major zymogen granule membrane glycoprotein 2 (GP2), *Escherichia coli*, FimH, receptor, adhesion, intestinal cell lines

## Abstract

Pathogenic bacteria, such as enteropathogenic Escherichia coli (EPEC) and enterotoxigenic E. coli (ETEC), cause diarrhea in mammals. In particular, E. coli colonizes and infects the gastrointestinal tract via type 1 fimbriae (T1F). Here, the major zymogen granule membrane glycoprotein 2 (GP2) acts as a host cell receptor. GP2 is also secreted by the pancreas and various mucous glands, interacting with luminal type 1 fimbriae-positive E. coli. It is unknown whether GP2 isoforms demonstrate specific E. coli pathotype binding. In this study, we investigated interactions of human, porcine, and bovine EPEC and ETEC, as well as commensal E. coli isolates with human, porcine, and bovine GP2. We first defined pathotype- and host-associated FimH variants. Second, we could prove that GP2 isoforms bound to FimH variants to various degrees. However, the GP2-FimH interactions did not seem to be influenced by the host specificity of E. coli. In contrast, soluble GP2 affected ETEC infection and phagocytosis rates of macrophages. Preincubation of the ETEC pathotype with GP2 reduced the infection of cell lines. Furthermore, preincubation of E. coli with GP2 improved the phagocytosis rate of macrophages. Our findings suggest that GP2 plays a role in the defense against E. coli infection and in the corresponding host immune response.

**IMPORTANCE** Infection by pathogenic bacteria, such as certain Escherichia coli pathotypes, results in diarrhea in mammals. Pathogens, including zoonotic agents, can infect different hosts or show host specificity. There are Escherichia coli strains which are frequently transmitted between humans and animals, whereas other Escherichia coli strains tend to colonize only one host. This host specificity is still not fully understood. We show that glycoprotein 2 is a selective receptor for particular Escherichia coli strains or variants of the adhesin FimH but not a selector for a species-specific Escherichia coli group. We demonstrate that GP2 is involved in the regulation of colonization and infection and thus represents a molecule of interest for the prevention or treatment of disease.

## INTRODUCTION

Zymogen granule membrane glycoprotein 2 (GP2) is a microbiota-sensing and immune-modulating molecule that is expressed at different sites in mammals, such as humans, cattle, pigs, and others (1–3). It is the most abundant protein in the pancreatic secretory granule membrane (4). During pancreatic secretion, GP2 is cleaved from the secretory granule membrane and released into the intestine. Furthermore, a variety of cells are able to express GP2, including M cells in the intestine; cells of various mucous glands in the digestive, respiratory, and genital tracts; and cells vital to mucosal innate and adaptive immune responses and enterocytes (3, 5–8). A number of alternative GP2 splice variants, which vary mainly in size (long and short isoforms), are expressed (9). So far, four isoforms in humans, two each in pig and cattle, have been predicted (10).

Members of the bacterial family *Enterobacteriaceae* use GP2 as a host cell receptor for their adhesion, and FimH as the tip of type 1 fimbriae (T1F) of Salmonella sp. and Escherichia coli has been confirmed to be the adhesin molecule for GP2 binding (6, 11). T1F are one of the most common adhesive fimbriae in *Enterobacteriaceae*, particularly in Salmonella sp. and E. coli (12, 13). T1F are assembled by the chaperone-usher pathway and bear the adhesive subunit FimH on the top of the fimbrial shaft, which binds to receptors with oligosaccharides containing mannose residues (14, 15). Several studies have shown that serotype-associated FimH variants of Salmonella specialists (host restricted or host adapted) and Salmonella generalists (host unrestricted) differ significantly in receptor recognition or tropism to different tissue types (16, 17). In our recent study, we have confirmed that Salmonella serotype-specific FimH sequences determine the recognition of glycoproteins (18). This interaction also depends on the level of T1F expression. However, Salmonella FimH variants did not differ in their binding to GP2 of human and pig origin or other standard glycoproteins like RNase B or horseradish peroxidase (18).

E. coli, also mostly expressing T1F, is highly diverse regarding its pathotypes and virulence mechanisms. Intestinal pathogenic E. coli, such as enteropathogenic E. coli (EPEC) and enterotoxigenic E. coli (ETEC), cause diarrhea in many hosts. EPEC uses T1F as one of its initial adhesins to adhere to host intestinal epithelial cells (19). After adherence, it destroys enterocyte microvilli and forms pedestals, causing diarrhea in humans, calves, and pigs (20–22). ETEC expresses various virulence and colonization factors, including enterotoxins and T1F (23), leading to traveler’s diarrhea in humans (24) and diarrheal syndromes in calves and piglets (22, 25, 26). E. coli can be transmitted between host species, and animals are often carriers of pathogenic E. coli which causes gastrointestinal diseases in humans (27). E. coli was also described as the cause of inflammatory bowel disease in humans. Notably, autoantibodies to GP2 have already been discussed as biomarkers for Crohn’s disease (CD)—an inflammatory bowel disease with the suspected involvement of E. coli (28–30).

In the present study, we have determined the interactions of GP2 of bovine (not studied so far), porcine, and human origin with diarrheagenic E. coli pathotypes EPEC and ETEC and commensal E. coli strains isolated from these particular hosts. First, we have recombinantly expressed two isoforms of GP2 of cattle, pig, and human in Sf9 insect cells as well as in respective host intestinal epithelial cells. Second, a collection of E. coli isolates were characterized regarding their FimH sequences. Subsequently, protein-protein interactions of FimH variants with GP2 isoforms were analyzed using enzyme-linked immunosorbent assay (ELISA) and surface plasmon resonance (SPR). Finally, adhesion of E. coli to recombinant and cell surface-expressed GP2, as well as effects of opsonization of E. coli by GP2 on host-cell adhesion and phagocytosis, were studied.

## RESULTS

### *fimH* gene sequences.

When 180 E. coli isolates were tested by PCR, *fimH* was not detected in 12 porcine and human ETEC or in 2 commensal E. coli, while 166 isolates (92.2%) carried this gene. Amplicon sequencing for all *fimH*-positive isolates resulted in the detection of 60 *fimH* alleles with 88 variable nucleotide sites and 30 amino acid sequence variants of FimH with 26 variable sites (Table 1; see Table S1 and S2 in the supplemental material). All *fimH* alleles and all amino acid sequences were present in the appropriate databases (CHTyper [https://cge.cbs.dtu.dk/services/chtyper/], Enterobase [https://enterobase.warwick.ac.uk/species/index/ecoli]; and NCBI GenBank). Several FimH variants occurred that were group specific; FimH variant 9 (*n* = 20 isolates; FimH variant designation please see Table 1) was carried only by ETEC, variant 7 (*n* = 5) only by EPEC, and variant 8 (*n* = 10) only by human E. coli isolates (Table 1).

**TABLE 1:** FimH variants^*a*^

FimH variant	No. of isolates	Amino acid in the FimH variant at the following position in K-12																									
		Signal peptide		Lectin domain														Pilin domain									
		-16	-12	10	25	27	62	66	70	75	78	82	106	119	128	143	153	163	166	195	202	230	234	242	244	269	273
		T	V	A	A	V	S	G	N	V	S	Y	A	A	V	Q	D	V	R	Y	A	T	A	A	G	Q	G
Variant 1	18	N	.	.	.	A	.	.	.	.	.	.	.	V	.	.	.	.	.	.	.	.	.	.	.	.	A
Variant 2	66	.	.	.	.	A	.	.	.	.	.	.	.	.	.	.	.	.	.	.	.	.	.	.	.	.	.
Variant 3	4	.	.	.	.	.	.	.	.	.	.	.	.	.	.	.	.	.	.	.	.	.	.	.	.	.	.
Variant 4	8	.	.	.	.	A	.	S	.	.	.	.	.	.	.	.	.	.	.	.	V	.	.	.	.	.	.
Variant 5	2	.	.	.	.	A	.	.	.	.	.	.	.	.	.	.	.	.	.	.	V	.	.	.	.	.	.
Variant 6	5	N	I	.	.	A	.	.	.	.	.	.	.	V	.	.	.	.	.	.	.	.	.	.	.	.	.
Variant 7	5	.	.	.	.	A	.	.	S	.	.	.	.	.	.	.	.	.	.	.	.	.	.	.	.	.	.
Variant 8	10	.	.	.	.	A	.	.	S	.	N	.	.	.	.	.	.	.	.	.	.	.	.	.	.	.	.
Variant 9	20	N	.	.	.	A	.	.	.	.	.	.	.	V	.	.	.	.	.	.	.	.	.	.	.	.	.
Variant 10	1	.	.	.	.	A	.	.	.	.	.	.	.	.	.	.	.	.	.	.	.	.	.	.	.	K	.
Variant 11	2	.	.	.	.	A	.	.	.	.	.	.	.	.	.	.	.	.	H	.	.	.	.	.	.	.	.
Variant 12	2	N	.	.	.	A	.	.	.	.	.	.	.	V	.	.	.	.	.	.	.	.	T	.	.	.	A
Variant 13	4	.	.	.	.	T	.	.	.	.	.	.	.	.	.	.	.	.	.	.	.	.	.	.	.	.	.
Variant 14	3	.	.	.	.	A	.	S	.	.	.	D	.	.	.	.	.	.	.	.	V	.	.	.	.	.	.
Variant 15	1	.	.	.	.	A	A	.	.	.	.	.	.	.	.	.	.	.	.	.	.	.	.	.	.	.	.
Variant 16	1	.	.	.	.	.	.	.	S	.	N	.	.	.	.	.	.	.	.	.	.	.	.	.	.	.	.
Variant 17	1	.	.	.	.	A	.	.	.	E	.	.	.	.	.	.	.	.	.	.	.	.	.	.	.	.	.
Variant 18	1	.	.	.	.	A	.	.	.	.	.	.	V	.	.	.	.	.	.	.	.	.	.	.	.	.	.
Variant 19	1	.	.	.	.	A	.	.	.	.	N	.	.	V	.	.	.	.	.	.	.	.	.	V	.	.	.
Variant 20	1	.	.	.	.	A	.	.	.	.	N	.	.	.	.	.	.	.	.	.	.	.	.	V	.	.	.
Variant 21	1	N	.	.	.	A	.	.	.	.	.	.	.	.	.	.	.	I	.	.	.	A	.	.	.	.	.
Variant 22	1	.	.	.	.	A	.	.	.	.	.	.	.	.	M	.	.	.	.	.	.	.	.	.	E	.	.
Variant 23	1	.	.	.	.	A	.	S	S	.	N	.	.	.	.	.	.	.	.	.	.	.	.	.	.	.	.
Variant 24	1	.	.	V	.	A	.	.	.	.	.	.	.	.	.	.	.	.	.	.	.	.	.	.	.	.	.
Variant 25	1	.	.	.	T	A	.	.	.	.	.	.	.	.	.	.	.	.	.	.	.	.	.	.	.	.	.
Variant 26	1	.	.	.	.	A	.	.	.	.	.	.	.	.	.	.	N	.	.	.	.	.	.	.	.	K	.
Variant 27	1	.	.	.	.	A	.	.	.	.	.	.	.	.	.	.	.	.	.	F	.	.	.	.	.	.	.
Variant 28	1	N	.	.	.	A	.	.	.	.	.	.	.	.	.	.	.	.	.	.	.	.	.	.	.	.	.
Variant 29	1	N	.	.	.	A	.	.	.	.	.	.	.	.	.	.	.	.	.	.	V	.	.	.	.	.	.
Variant 30	1	.	.	.	.	A	.	.	.	.	.	.	.	.	.	L	.	.	.	.	.	.	.	.	.	.	.

a FimH variants were aligned with FimH of E. coli strain K-12 substr. MG1655. Only variable amino acid sites with the corresponding positions are shown. A point represents the same amino acid like strain K-12.

### Expression and characterization of bovine GP2 isoforms.

**FIG 1:**
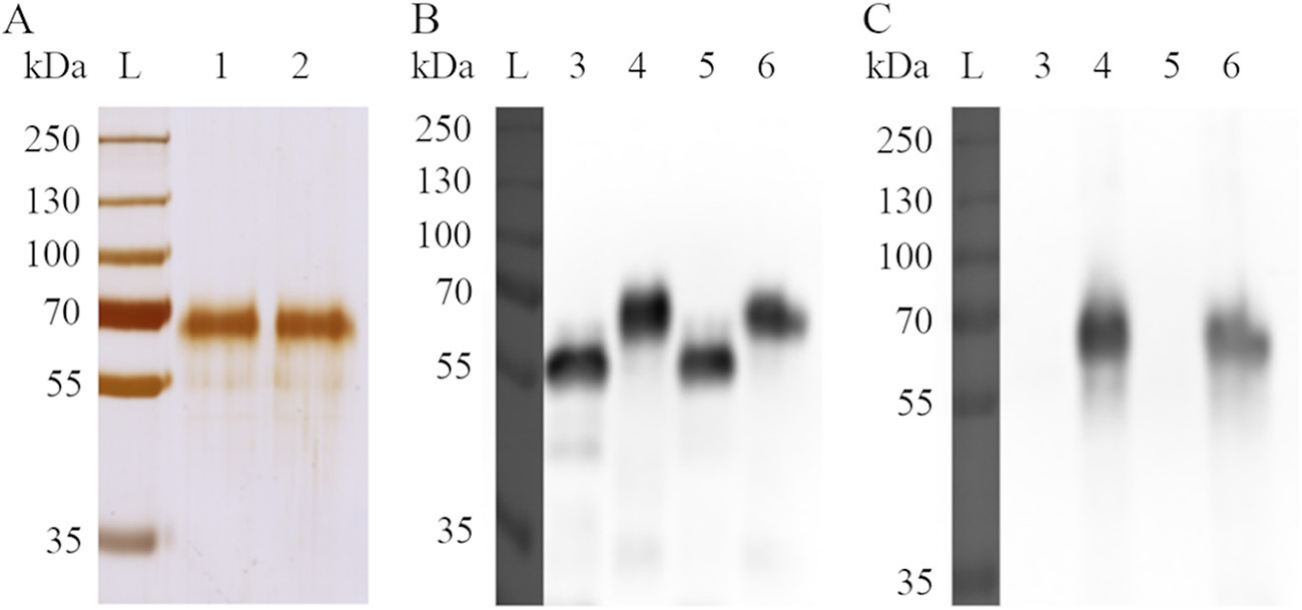
Expression of bovine GP2 and its deglycosylation. (A) Silver staining of GP2; lane L: protein ladder; lane 1: bovine GP2 isoform 1, 535 amino acids (GP2#Bo1); lane 2: bovine GP2 isoform 2, 534 amino acids (GP2#Bo2). (B) Western blotting with anti-6-His antibody of deglycosylated and glycosylated GP2; lane 3: deglycosylated GP2#Bo1; lane 4: glycosylated GP2#Bo1; lane 5: deglycosylated GP2#Bo2; lane 6: glycosylated GP2#Bo2. (C) Western blotting with ConA of deglycosylated and glycosylated GP2; lane 4: glycosylated GP2#Bo1; lane 6: glycosylated GP2#Bo2.

Based on mRNA isolation from bovine pancreatic tissue and cDNA analysis, we defined two bovine GP2 sequences which were confirmed by Sanger sequencing and found identical to the already available cDNA sequences in databases. Both bovine GP2 isoforms belonged to the long GP2 isoform (like also porcine GP2 isoform 1 [GP2#Su1] and human GP2 isoform 2 [GP2#Ho2]) and consisted of 534 (isoform 1, GP2#Bo1) and 535 amino acids (isoform 2, GP2#Bo2). Alignment of their amino acid sequences with human and porcine GP2 sequences revealed 75% and 78% identity with human and porcine GP2 isoforms, respectively, whereas the sequence similarity (amino acid exchange by an amino acid with similar properties) was 84% and 87%, respectively (see Fig. S1 in the supplemental material). Particularly, the epidermal growth factor (EGF)-like domain and zona pellucida (ZP) domains as well as the glycosylphosphatidylinositol (GPI) anchor showed the highest similarities with 86% to 100%. Both bovine GP2 isoforms were expressed in Sf9 insect cells and successfully purified (Fig. 1A). The glycosylation of proteins was confirmed by Western blotting with concanavalin A (ConA) (Fig. 1C). ConA, a lectin isolated from Jack beans (Canavalia ensiformis), specifically binds high mannose type, hybrid type, and complex type N-glycans (31). ConA bound well to recombinant bovine GP2, but after deglycosylation ConA, it did not bind to bovine GP2, confirming glycosylation of recombinant GP2. Both GP2 isoforms were deglycosylated under denaturing conditions since deglycosylation under native conditions had resulted in incomplete removal of carbohydrate residues in our previous studies on porcine GP2 (18). As expected, after Western blotting, glycosylated proteins appeared as larger proteins and deglycosylated proteins as smaller proteins (Fig. 1B and C).

**FIG 2:**
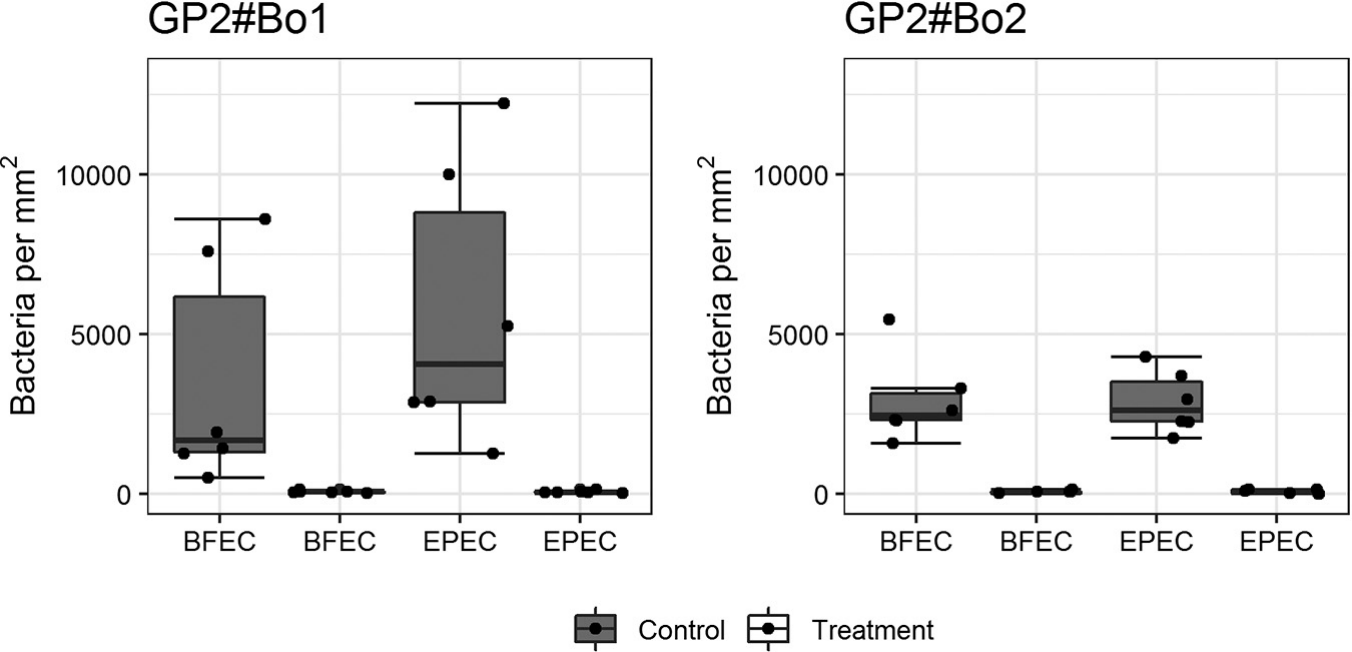
Glycosylation is necessary for binding of E. coli to bovine GP2. The E. coli was incubated in wells of a GP2-coated 96-well plate. The bound bacteria were fluorescence stained and analyzed with the VideoScan technology. Results are shown as the number of bacteria attached per mm^2^ of the well bottom. Representatively, binding of one commensal E. coli (BFEC) and one EPEC isolate of bovine origin is shown. GP2 isoforms were deglycosylated under denaturing conditions (treatment). Control GP2 (control) was exposed in parallel to same denaturing conditions but not treated with the enzyme (still glycosylated). The experiment was performed three times in duplicates. Data are presented as box-whisker plots. The dots represent the medians of the duplicates performed three times. GP2#Bo1, bovine GP2 isoform 1; GP2#Bo2, bovine GP2 isoform 2.

An analysis of 45 E. coli isolates of bovine, human, and porcine origin in the GP2 assay substantiated that the glycosylation of GP2 was essential for E. coli binding since each isolate bound to glycosylated GP2 but not to deglycosylated GP2 which is exemplarily shown for one isolate in Fig. 2.

### Correlation between FimH expression and binding to GP2.

**FIG 3:**
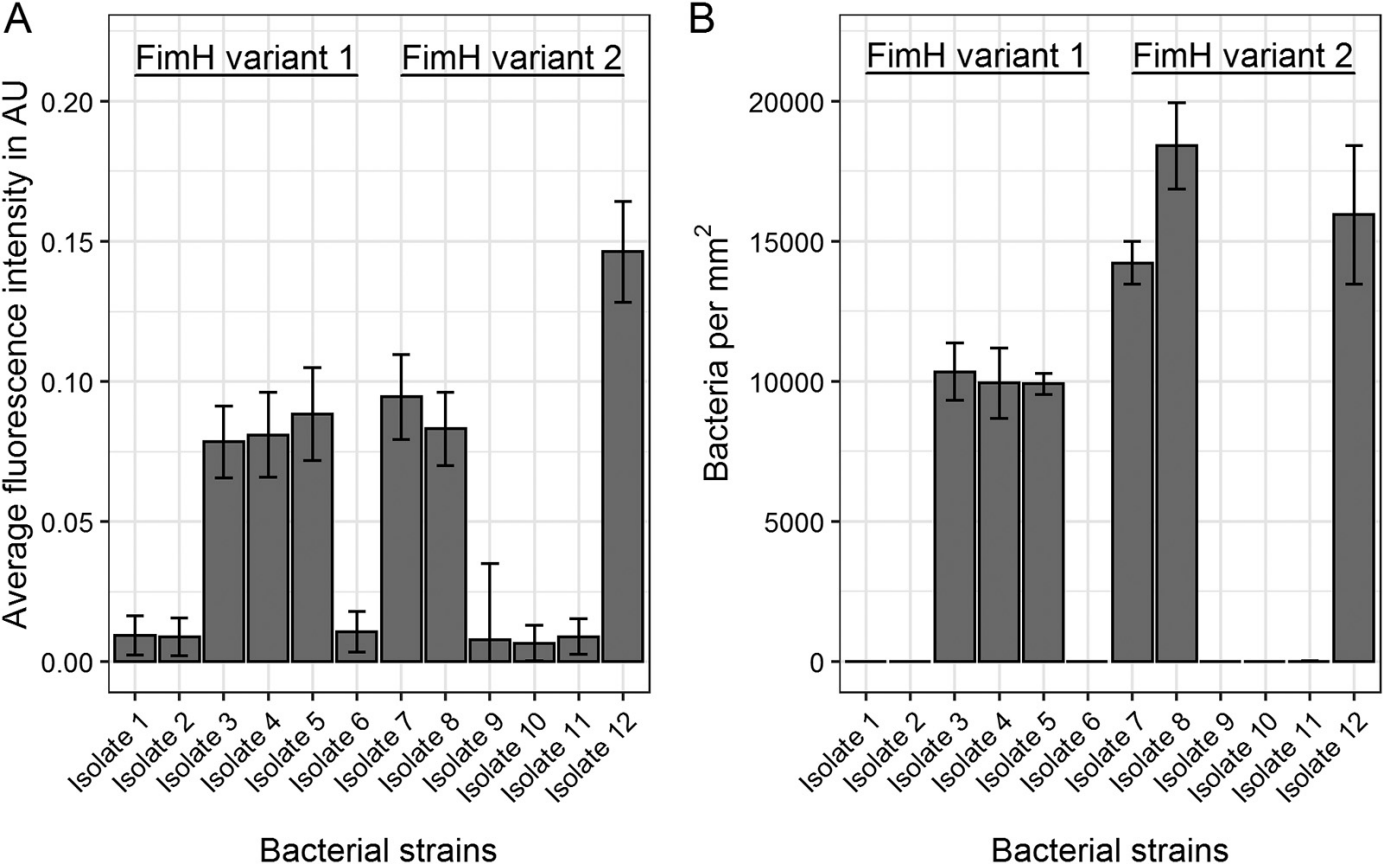
Correlation of FimH expression with binding to bovine GP2. (A) FimH expression; bacteria were stained with a primary anti-FimH antibody and a fluorescent secondary anti-rabbit IgG antibody followed by quantification of the fluorescence by the VideoScan technology (= fluorescence intensity). (B) GP2 assay; bacteria were incubated on GP2#Bo1, and the fluorescence-stained adherent bacteria were counted using the VideoScan technology (= bacteria bound to 1 mm^2^ of the well bottom coated with GP2). Data are shown as median with average deviation of median of the three times triplicate determination.

A total of 12 E. coli isolates carrying 1 of 2 FimH variants (variants 1 and 2) were selected and submitted to 2 assays in parallel for quantifying their FimH expression and their binding capacities to bovine GP2 (GP2 assay). FimH expression was tested by staining bacteria with anti-FimH antibodies. E. coli isolates with the same FimH variant differentially expressed FimH; some isolates expressed FimH, while others did not. For both variants, FimH expression was strongly correlated with bacterial binding to GP2, as the E. coli isolates bound to GP2 only if they also expressed FimH (Fig. 3).

**FIG 4:**
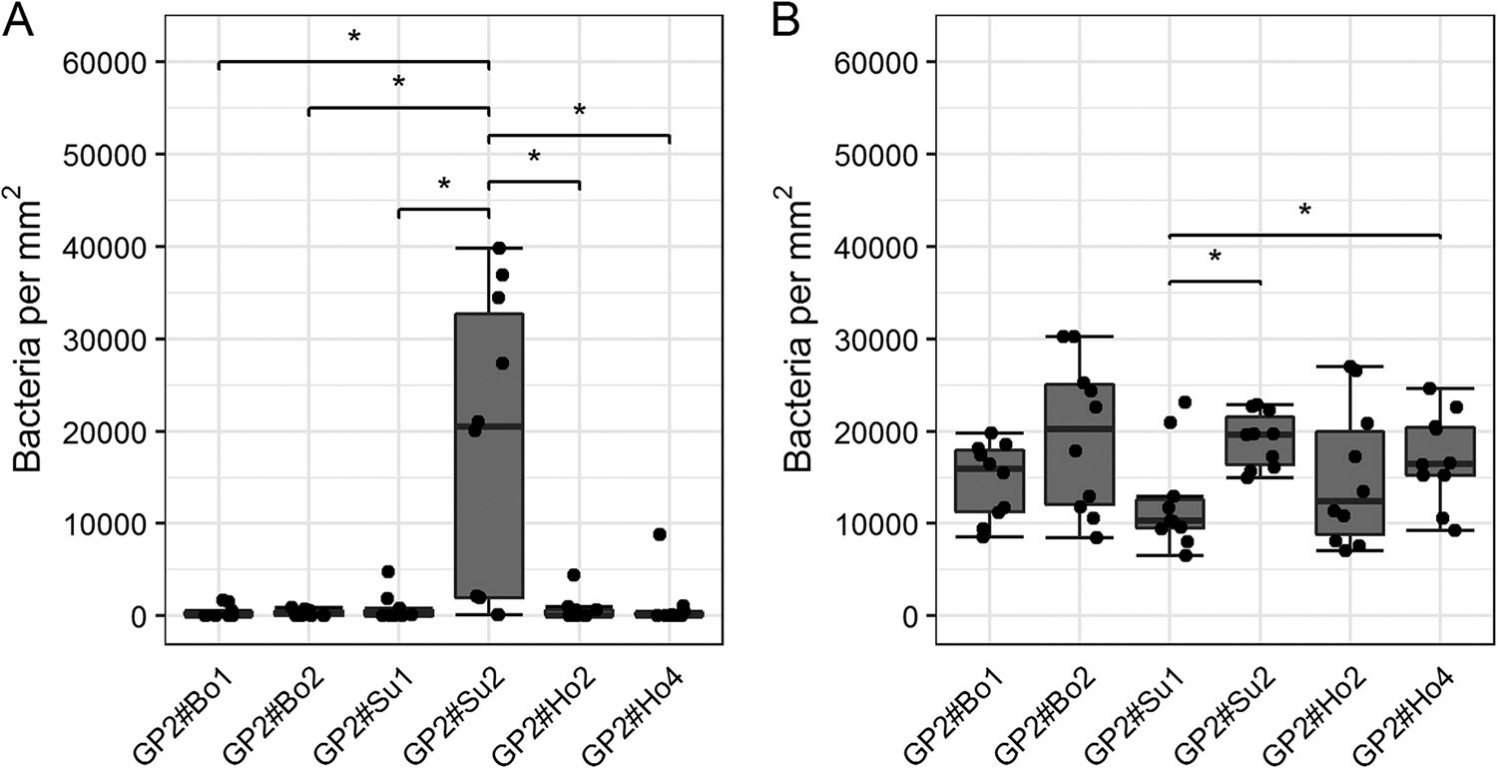
Specific binding of E. coli isolates to GP2 isoforms. Two representative isolates are shown, as follows: porcine commensal E. coli (FimH variant 6) better adhered to one porcine isoform (A) and a human commensal E. coli (FimH variant 8) with less adherence to one porcine isoform (B). Significant differences of medians are illustrated by stars (*) with a *P* value of <0.05. The data are presented as box-whisker plots. The dots represent the medians of the duplicates performed five times. GP2#Bo1, bovine GP2 isoform 1; GP2#Bo2, bovine GP2 isoform 2; GP2#Su1, porcine GP2 isoform 1; GP2#Su2, porcine GP2 isoform 2; GP2#Ho2, human GP2 isoform 2; GP2#Ho4, human GP2 isoform 4.

**FIG 5:**
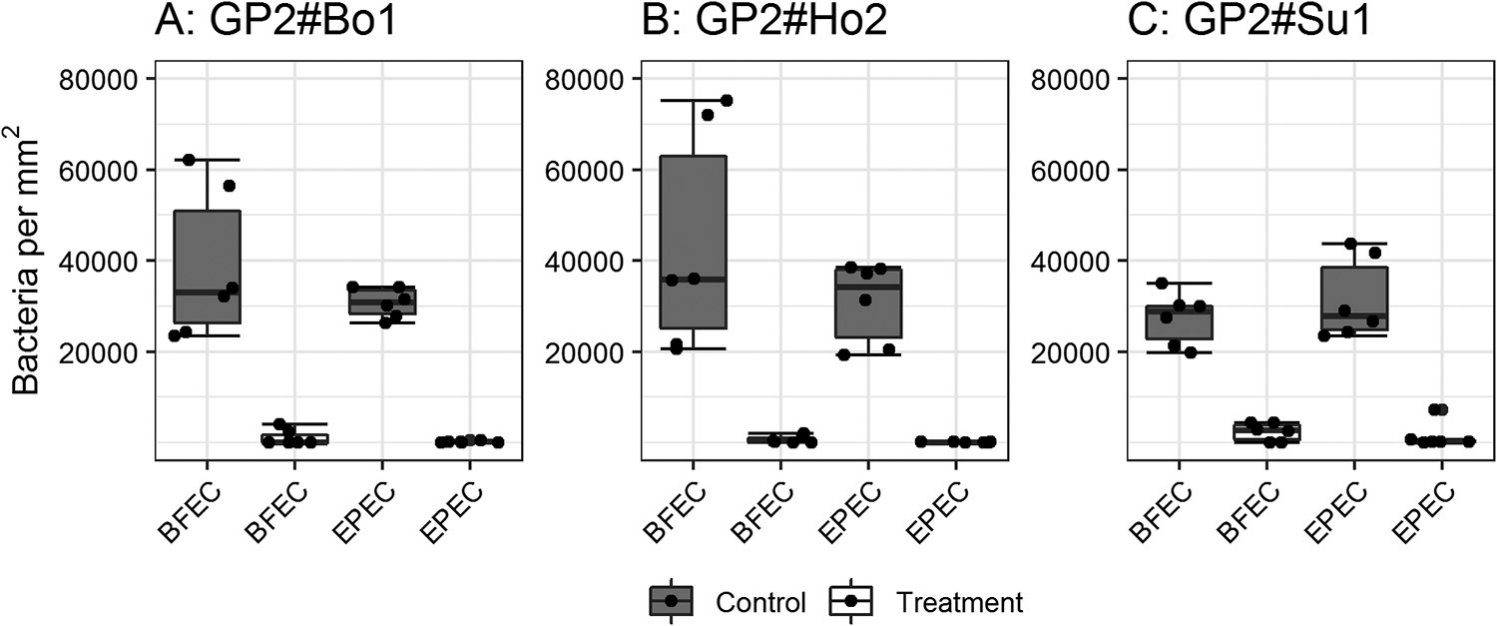
Blocking of bacterial binding to GP2 by d-mannose. The representative bacteria, a commensal E. coli (BFEC, FimH variant 1) and a bovine ETEC (FimH variant 9), were incubated for 1 h with d-mannose (treatment). For a control, the assay was performed under the same conditions but without a sugar preincubation (control). The binding behavior was investigated with the bovine GP2 isoform 1 (A), the human GP2 isoform 2 (B), and the porcine GP2 isoform 1 (C). Data are presented as box-whisker plots. The dots represent the medians of the duplicates performed three times.

Using GP2 assays, we tested for host-specific GP2 binding of bacteria. EPEC, ETEC, and commensal E. coli isolates, when incubated with bovine, porcine, and human GP2 isoforms, defined 4 binding types, as follows: no (<1,000 bacteria per mm^2^), low (1,000 –to 9,999 bacteria per mm^2^), medium (10,000 to 39,999 bacteria per mm^2^), and high binding (>40,000 bacteria per mm^2^). Although single strains bound differently to GP2 isoforms, no E. coli group bound in a host-specific manner; e.g., bovine isolates did not bind better to bovine GP2. Out of 180 isolates, 137 isolates bound to GP2. Of these 137 isolates, 14 isolates (belonging to FimH variants 1, 2, 6, 8, 9, and 29) bound to 1 GP2 isoform significantly better than to another isoform, as validated by repeating the assay 5 times (Fig. 4). The other 123 isolates bound similarly to all GP2 isoforms. The 43 nonbinding isolates were mainly ETEC (24/43), including the 12 ETEC lacking the *fimH* gene. FimH-positive ETEC had the worst binding to GP2 compared with that of the other pathotypes (77%). The two commensal isolates lacking the *fimH* gene were low binding, showing that isolates can express other adhesins binding to GP2. Additionally, differences in adhesion to GP2 could be seen when comparing the bacterial origin. A total of 93% of FimH-positive E. coli isolates from bovine adhered to GP2, while only 79% of human isolates and 69% porcine isolates bound to GP2. When an isolate bound well to GP2, then it also bound well to another glycoprotein, such as RNase B, indicating a general similar binding property to different glycoproteins (see Fig. S2 in the supplemental material). In contrast, an isolate which bound well to glycoproteins did not bind to nonglycoproteins like bovine serum albumin (BSA), showing that the carbohydrate residues are receptor molecules of GP2 (Fig. S2). Results were confirmed by blocking the binding of bacteria to mannose residues of GP2 with d-mannose. d-Mannose blocked the binding of 45 representative isolates to the 3 long isoforms by 79% (bovine and human GP2) and 64% (porcine GP2) on average (Fig. 5). In contrast, d-glucose did not block the binding (data not shown).

### Characterization of GP2-FimH interactions by ELISA and SPR analysis.

**FIG 6:**
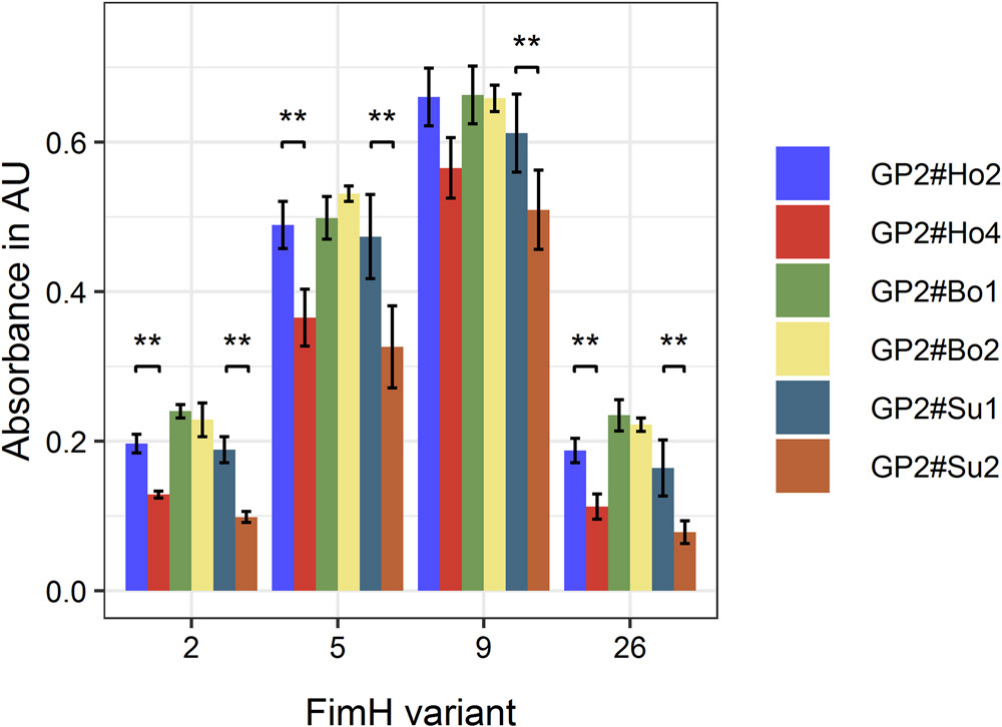
Specific binding of FimH variants to GP2 isoforms. ELISA with four FimH variants and six GP2 isoforms. Experiments were performed three times in duplicates. The FimH variants bound best to the long GP2 isoforms. The GP2-FimH interaction depends on both the FimH variant and the GP2 isoform. **, significant differences with *P* values of <0.01 were found between the long and the short GP2 isoforms of one host. Data shown are median and the average deviation of median. GP2#Ho2, human GP2 isoform 2; GP2#Ho4, human GP2 isoform 4; GP2#Bo1, bovine GP2 isoform 1; GP2#Bo2, bovine GP2 isoform 2; GP2#Su1, porcine GP2 isoform 1; GP2#Su2, porcine GP2 isoform 2. Long GP2 isoforms are as follows: GP2#Ho2, GP2#Bo1, GP2#Bo2, and GP2#Su1; short GP2 isoforms are as follows: GP2#Ho4 and GP2#Su2.

It is known that FimH variants bind to glycoproteins and intestinal epithelial cells depending upon their amino acid sequences (32–34). To exclude factors like FimH expression levels, we reduced our model to the pure FimH-GP2 interaction and included recombinantly expressed and purified FimH (variants 2, 5, 9, and 26) and GP2 isoforms (six isoforms). When GP2 isoforms were immobilized on 96-well plates for ELISA, the FimH proteins bound differently to the GP2 isoforms (Fig. 6), with FimH variant 9 binding best. Consistently, all FimH proteins bound better to the long GP2 isoforms (GP2#Bo1, GP2#Bo2, GP2#Su1, and GP2#Ho2) than to the short isoforms (GP2#Su2 and GP2#Ho4). As shown in Fig. 6, the FimH amino acid sequence variation indeed affected the binding to GP2.

To assess the affinity between FimH and GP2 and to test FimH/GP2 interaction under flow conditions, the association rate constant (*k_a_*) and the dissociation rate constant (*k_d_*) were determined for each FimH-GP2 pair using the 1:1 Langmuir model after SPR measurements. When we immobilized GP2 on CM5 chips, a global fitting of measured values was not possible, as sensorgrams showed strong deviations in association and dissociation compared with those of other studies (16, 35). Therefore, the calculation of the *k_d_*/*k_a_* ratio was not possible.

**FIG 7:**
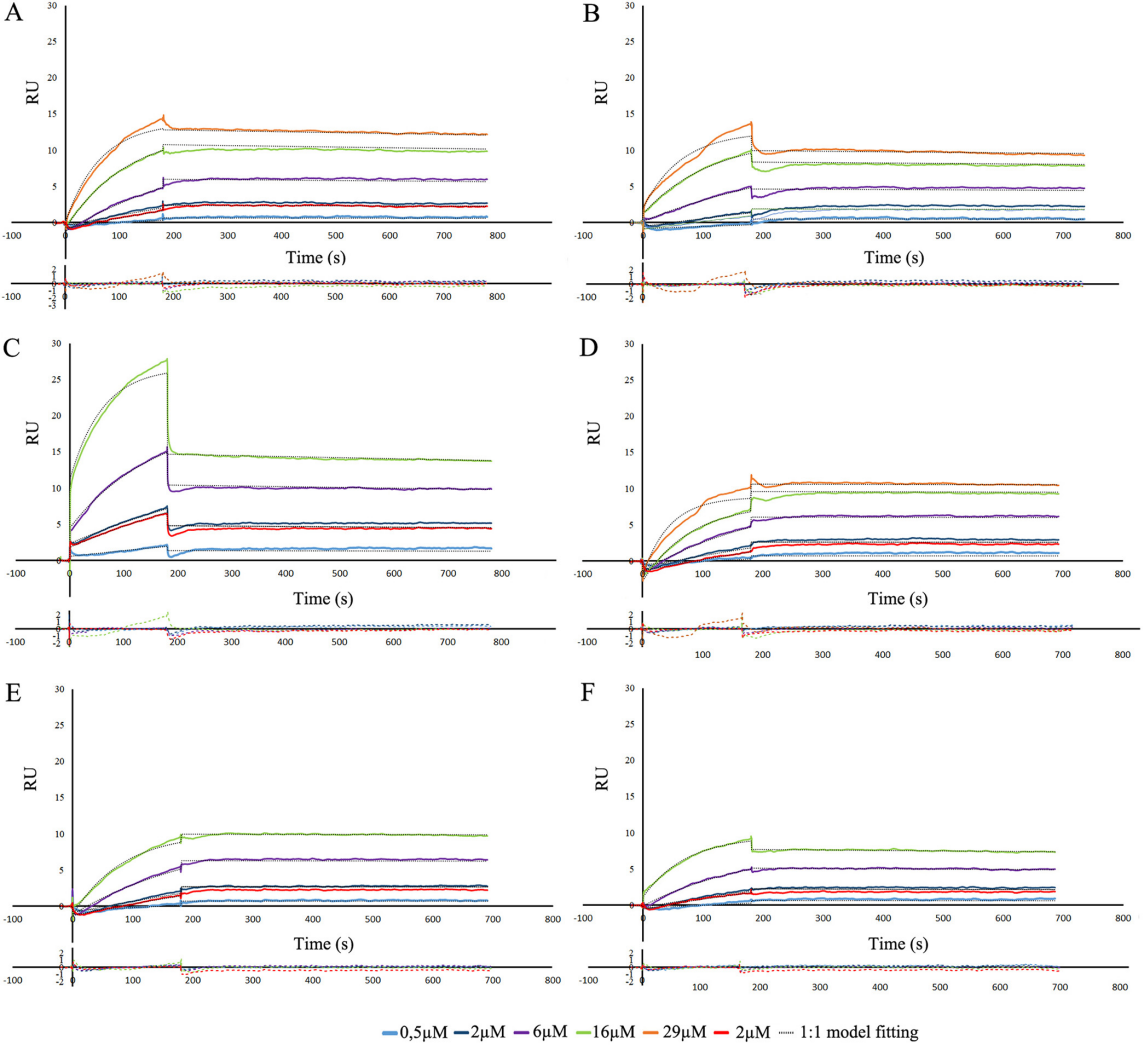
Interaction of various GP2 isoforms (GP2#Bo1 [A], GP2#Bo2 [B], GP2#Ho2 [C], GP2#Ho4 [D], GP2#Su1 [E], and GP2#Su2 [F]) with immobilized FimH variant 5 analyzed with an SPR. Lines represent different concentrations of the analyte (0.5, 2.0, 6.0, 16, and 29 μM) in PBS-*P*+ buffer. The analyte concentration of 2.0 μM was analyzed twice to ensure proper measurement. Binding data were collected at a flow rate of 30 μL/minutes. Below each sensorgram, residual plots are presented, defining differences between experimental data and fitted kinetic model.

Alternatively, we immobilized FimH variants with the highest binding in ELISA (variants 5 and 9) on CM5 chips and studied binding of all six GP2 isoforms. Association and dissociation rate constants were calculated for each pair using global 1:1 Langmuir fitting (Table 2). The sensorgrams are shown in Fig. 7 and Fig. S3 in the supplemental material. In the case of FimH variant 5, GP2#Ho4 and GP2#Su1 bound significantly stronger with a *k_d_* of around 4 to 8 times lower than that of all other GP2 isoforms. FimH variant 9 had similar values of association and dissociation with most of GP2 isoforms. This FimH variant bound four times stronger to GP2#Ho2 and seven times weaker to GP2#Su1 than the FimH variant 5. Additionally, the interaction between FimH variant 9 and GP2#Ho4 was very strong, which was around 23 times stronger than that using the FimH variant 5. This high binding was caused mainly by a very low dissociation rate.

**TABLE 2:** Calculated *k_d_* values of the FimH-GP2 interactions^*a*^

Interaction	*k_a_* (1/M·s)	*k_d_* (1/s)	*K_D_* (M)	*R*_max_ (RU)	Chi² (RU²)
FimH variant 5 with:					
GP2#Bo1	5.38E+02	9.65E-05	1.79E-07	13.74	0.337
GP2#Bo2	5.45E+02	7.41E-05	1.36E-07	10.61	0.309
GP2#Ho2	1.04E+03	1.02E-04	9.78E-08	15.54	0.346
GP2#Ho4	7.72E+02	1.68E-05	2.17E-08	10.82	0.308
GP2#Su1	7.58E+02	1.71E-05	2.26E-08	11.28	0.213
GP2#Su2	8.70E+02	8.32E-05	9.57E-08	8.447	0.122
FimH variant 9 with:					
GP2#Bo1	5.51E+02	1.87E-04	3.40E-07	21.91	0.558
GP2#Bo2	6.12E+02	5.72E-05	9.34E-08	12.47	0.22
GP2#Ho2	1.06E+03	2.38E-05	2.25E-08	17.35	0.117
GP2#Ho4	4.65E+02	4.31E-07	9.27E-10	13.67	0.43
GP2#Su1	5.35E+02	8.44E-05	1.58E-07	17.33	0.233
GP2#Su2	8.98E+02	8.84E-05	9.85E-08	11.95	0.214

a *K_D_*, *k_d_*/*k_a_* ratio; *R*_max_, maximal response units.

### GP2 expression in GP2-transduced FBJ cells.

**FIG 8:**
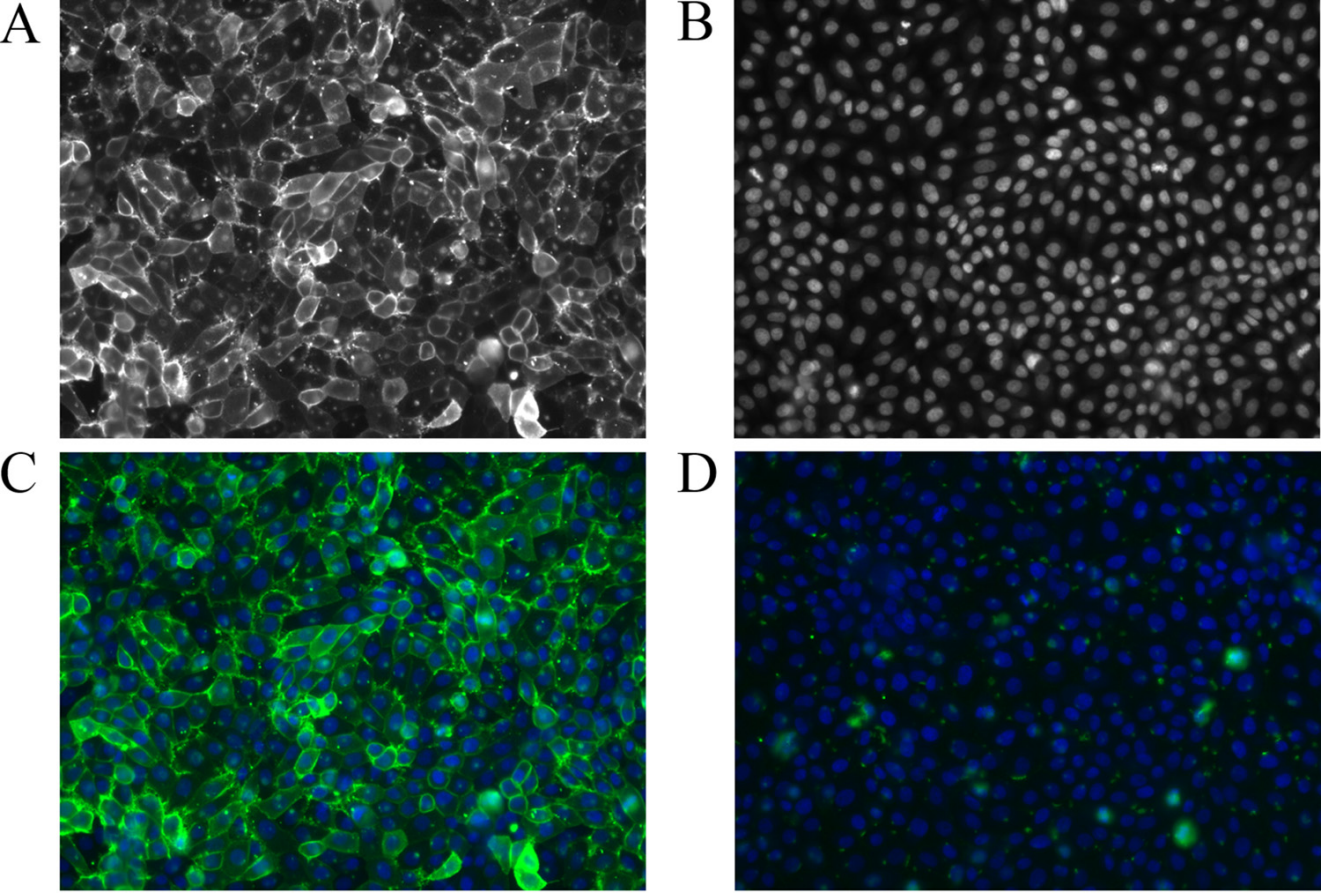
GP2 expression on the surface of FBJ cells. The cells were stained with rabbit anti-GP2 antibody serum (1:250) and secondary goat antibody anti-rabbit IgG Alexa Fluor 647 and DAPI. (A) GP2 staining = GP2 expression. (B) DAPI staining of cell nuclei. (C) Merge of A and B showing the FBJ cell line expressing bovine GP2 isoform 2 (FBJ-GP2#Bo2). (D) FBJ cell line transfected with empty vector as negative control. GP2 expression was similar for bovine GP2 isoform 1 (FBJ-GP2#Bo1).

The two bovine GP2 isoforms 1 and 2 were transduced by lentiviruses into the fetal bovine intestinal epithelial cell line FBJ. A negative control was included using the empty plasmid/lentivirus DNA without the GP2 sequence. Expression of GP2 on cell surfaces was ensured by retaining the GPI anchor during genetic engineering. Expression of GP2 was confirmed initially by quantitative PCR (qPCR) at the mRNA level (data not shown). Second, indirect immunofluorescence (IIF) confirmed the expression of the GP2 on cell surfaces at the protein level (Fig. 8). Thus, transduction was successful and both GP2 isoforms were expressed on the cell surface. A human (LoVo) and a porcine intestinal epithelial cell line (IPEC-J2) transduced for GP2 expression included in the following experiments were already created in previous studies.

### Adhesion assays.

Adhesion of 90 E. coli isolates (10 isolates from each pathotype and host) was tested on GP2-expressing cells, including bovine intestinal epithelial cells FBJ-GP2#Bo1 and #Bo2 (bovine isoforms 1 and 2 [this study]), human intestinal epithelial cells LoVo-GP2-#Ho1 and #Ho2 (human isoforms 1 and 2 [this study]), and porcine intestinal epithelial cells IPEC-J2-GP2#Su1 and #Su2 (porcine isoforms 1 and 2 [18]). Cells with the empty plasmid were used as controls (no GP2 expression).

**FIG 9:**
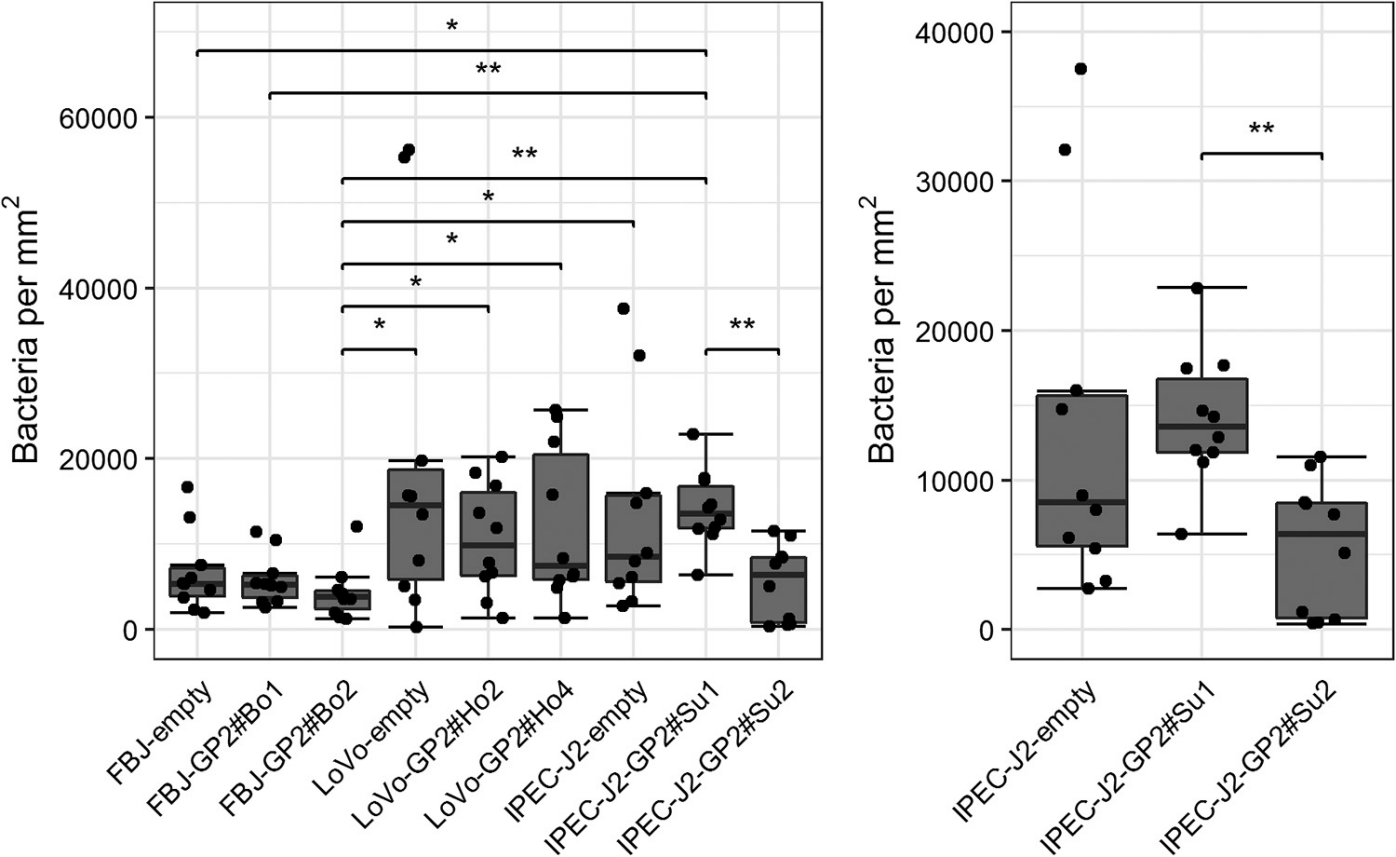
Specific binding of isolates to cell lines. One representative isolate of total 90 E. coli, a bovine EPEC (FimH variant 15), is shown. The binding to the porcine cell lines is highlighted in the right image. Significant differences are illustrated by stars (* or **) with *P* values of <0.05 (*) and <0.01 (**). The data are presented as box-whisker plots. The dots represent the medians of the duplicates performed five times. FBJ/LoVo/IPEC-J2-empty, no GP2 expression on FBJ//LoVo/IPEC-J2 cell lines; FBJ-GP2#Bo1/Bo2, bovine GP2 isoform 1/2 expressed on FBJ cell line; LoVo-GP2#Ho2/Ho4, human GP2 isoform 2/4 expressed on LoVo cell line; IPEC-J2-GP2#Su1/Su2, porcine GP2 isoform 1/2 expressed on IPEC-J2 cell line.

We again defined four binding types, as following: no (<500 bacteria per mm^2^), low (500 to 4,999 bacteria per mm^2^), medium (5,000 to 19,999 bacteria per mm^2^), and high binding (>20,000 bacteria per mm^2^). In general, we observed the same trend as that in the GP2 assays; an E. coli isolate that bound well to GP2 in the protein assay also adhered well in the adhesion assay. A total of 77 isolates (84%) bound significantly better/worse to one cell line than to another cell line. Additionally, all of these 77 isolates had at least 4 such differences. On average, isolates bound worst to FBJ cells and best to porcine IPEC-J2 cells (see Fig. S4 in the supplemental material). We had hypothesized that a higher number of bacteria would bind to GP2-expressing cell lines. However, numbers of cell-bound E. coli bacteria were not different between GP2-expressing and nonexpressing cell lines (Fig. 9).

**FIG 10:**
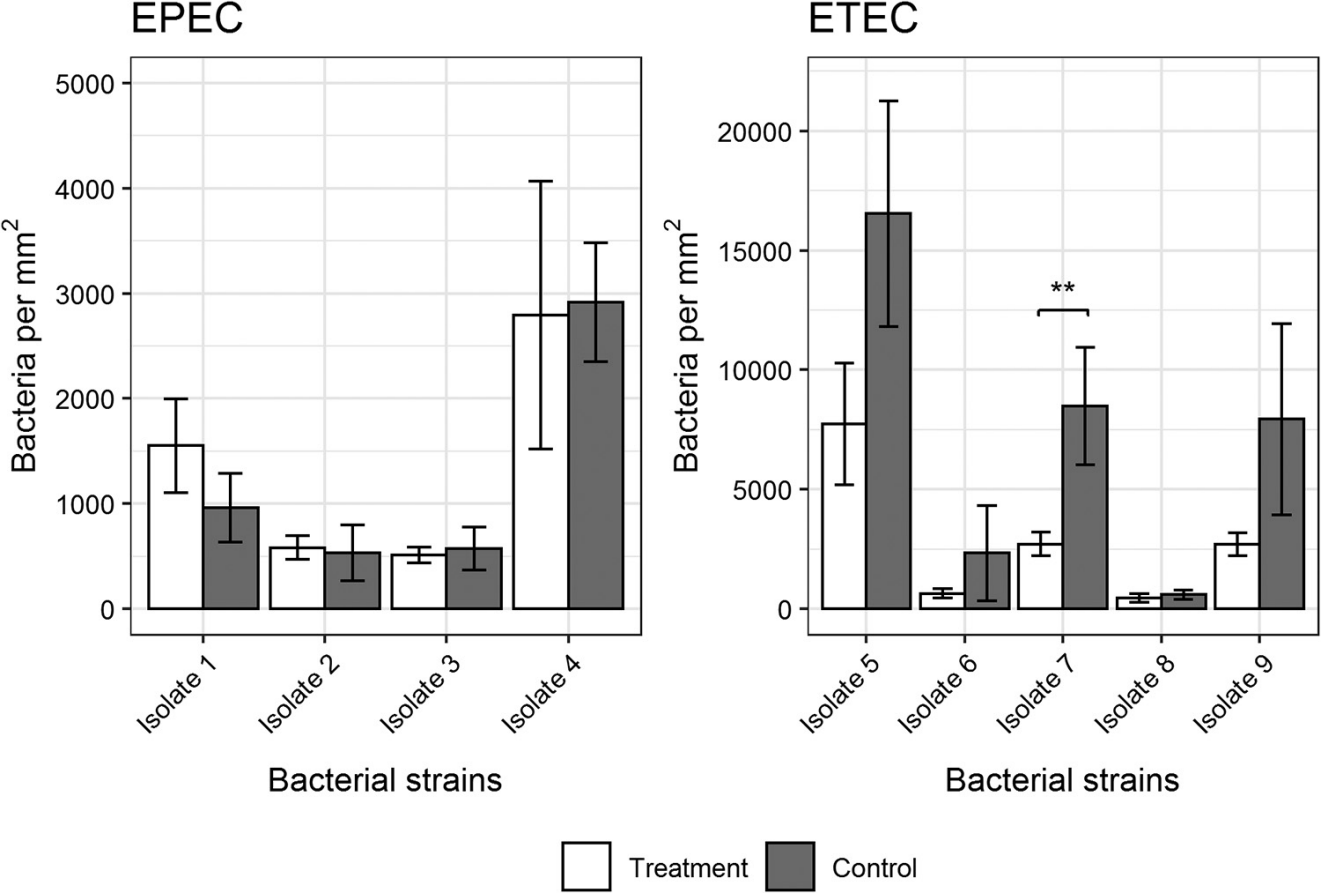
Effect of preincubation of 9 E. coli isolates with bovine GP2 isoform 1 on the binding to FBJ cell lines. Bacteria were incubated for 1 h with GP2 (treatment); additionally, an adhesion assay with FBJ cell lines was performed under same conditions without GP2 treatment (control). Shown are results of FBJ-GP2#Bo1 cells. Results of wild-type FBJ cells were similar. Significant differences in binding are illustrated by stars (**) with a *P* value of <0.01. The assay was performed three times in duplicates. Data shown are median and the average deviation of median.

We also tested whether preincubation of bacteria with GP2 affected the binding activity of selected EPEC (*n* = 4) and ETEC (*n* = 5) isolates to wild-type FBJ cells (no GP2 expression). This arrangement would reflect the situation in the intestine where bacteria are exposed to soluble GP2 released from the pancreas. Additionally, we used the generated GP2-expressing FBJ cell lines for these experiments. We preincubated the bacterial isolates with GP2 in a concentration of 10 μg/mL before exposing them to the FBJ cells. As shown in Fig. 10, preincubation of the ETEC isolates with GP2 decreased the number of bacteria bound to FBJ cells, independently of whether wild-type or GP2-transduced cells were used. In contrast, none of the EPEC isolates was affected by GP2 preincubation.

### Phagocytosis assay.

**FIG 11:**
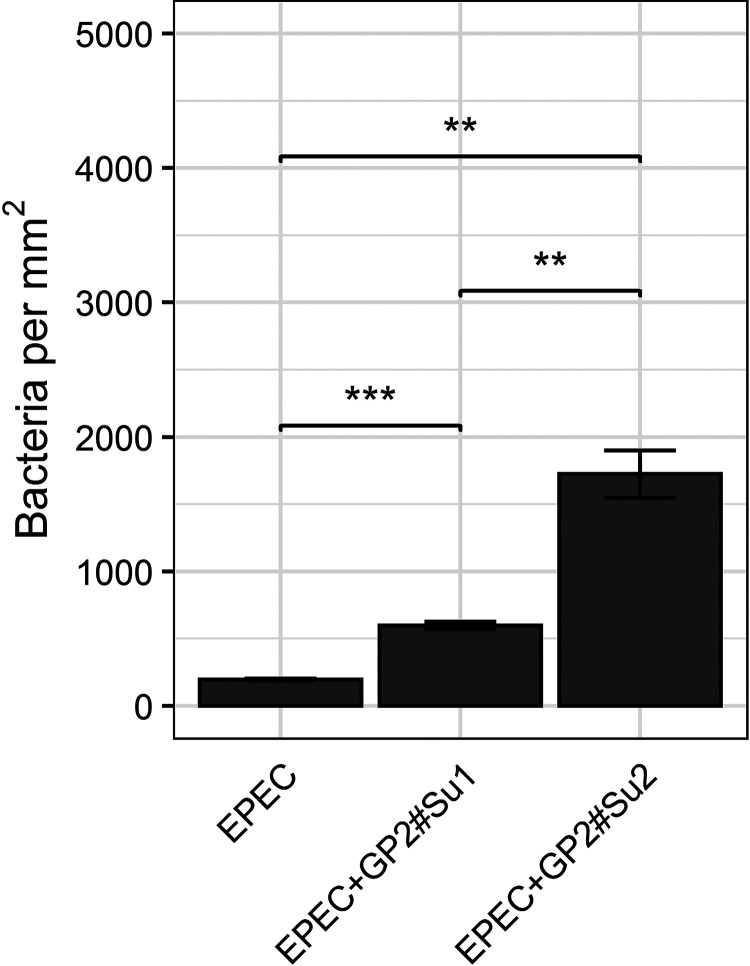
Phagocytosis of bacteria by porcine macrophages. A porcine EPEC was preincubated with porcine GP2 isoform 1 (GP2#Su1) and isoform 2 (GP2#Su2). After unbound GP2 was washed off, the bacteria were incubated with porcine macrophage cell line 3D4/31. The phagocytosed bacteria were fluorescently stained, counted, and presented as the number of bacteria per mm^2^ (of macrophage colonized area). Experiments were performed three times in triplicates. Data shown are mean and standard deviation. **, *P* ≤ 0.01; ***, *P* ≤ 0.001.

The results of the aforementioned experiments suggest that GP2 molecules exposed on the cell surface rarely affect binding of pathogenic E. coli to intestinal epithelial cells when expressed within an intact layer of other glycoproteins. However, freely available GP2 like that coming from the pancreas may have more significant effects on the infection process. In this context, we tested the influence of soluble GP2 on one of the first steps of a host immune response, such as bacterial phagocytosis by macrophages (36). We investigated in a phagocytosis assay whether bacterium-bound GP2 affects the phagocytosis rate of macrophages by using the porcine macrophage cell line 3D4/31 and the human macrophage cell line THP-1. We studied the phagocytosis of one porcine EPEC. Preincubation of bacteria with GP2 significantly increased phagocytic rates of both macrophage cell lines (Fig. 11). There was no difference between the effects of human and porcine GP2 on phagocytosis rates and no difference between the two cell lines 3D4/31 and THP-1 (data not shown). However, short GP2 isoforms stimulated a higher phagocytosis rate than long isoforms.

## DISCUSSION

GP2 acts as a receptor for the T1F adhesin FimH of bacteria whose FimH-GP2 interaction leads to innate or adaptive immune responses (3, 37). As GP2 binds T1F-positive E. coli (2, 11), we hypothesized that bacterial origins, pathotypes, and FimH variations as well as GP2 isoforms and host species determine the FimH-GP2 interaction.

The *fimH* gene was present frequently in our E. coli isolates, which is in accordance with other studies (38). The gene was least present in the group “porcine ETEC.” Other studies have shown that the occurrence of the *fimH* gene in E. coli in pigs can vary considerably and that certain strains do not need fimbriae to colonize the intestine (39, 40). FimH variant 2 was the predominant type in our study which has been also documented by several other authors (34, 41–43). In our study, several FimH variants were associated with EPEC and ETEC as well as with human isolates which was not mentioned so far. To date, only specific *fimH* mutations have been associated with adherent-invasive E. coli and E. coli from bovine, porcine, and avian origin (32, 44). Particularly, the exclusive and frequent occurrence of FimH variant 9 in 20 ETEC isolates was remarkable since the *fimH* gene was detected in only 80% of ETEC isolates. The human-specific FimH variant 8 was present in 4 EPEC and 6 commensals but not in ETEC. This finding suggests that different E. coli populations prefer different FimH variants for intestinal adhesion.

Our experiments confirmed previous studies demonstrating that T1F can bind to glycan residues of proteins which can be blocked by mannose (2, 17, 45). The FimH/T1F expression level strongly determined the binding of E. coli to GP2. This finding could reflect that FimH expression is tightly regulated and affected by the presence/expression of other adhesins or other factors. For example, in uropathogenic E. coli (UPEC), expression of pyelonephritis-associated pili (pap) suppressed the expression of T1F (46). Isolates lacking the *fimH* gene mostly did not bind to GP2. However, two of these isolates lacking the *fimH* gene still interacted with GP2, indicating other bacterial adhesins for GP2 which have to be determined in future studies.

We hypothesized that specific E. coli subpopulations defined by their host species of origin and/or pathotype bind to specific GP2 isoforms. The majority of E. coli isolates was able to bind to GP2. While 123 isolates bound similarly to all GP2 isoforms, 14 isolates displayed significantly different binding rates to at least 2 isoforms. Thus, some E. coli strains may prefer specific GP2 isoforms as the receptor in the intestine. *FimH*-positive ETEC bound generally worst to GP2 and glycoproteins. Although T1F has been described as an important adhesin for ETEC (23), T1F seems to be less important for ETEC than for, e.g., commensal E. coli.

To exclude interactions other than the pure GP2-FimH interaction, we performed ELISA and SPR experiments with four FimH variants and six GP2 isoforms. In general, FimH bound better to long GP2 isoforms, including both bovine isoforms, than to short isoforms. It is likely that additional asparagine residues in the long GP2 isoforms and accordingly more mannose residues offer additional binding sites for FimH. The four FimH variants bound with different amounts to GP2. FimH variant 9 bound most to GP2, followed by FimH variant 5. Single amino acid mutations in the FimH sequence can cause such binding differences (32–34). It is known that modification of a binding pocket in an adhesin influences the adhesion of the respective bacterium to host cell receptors (47). However, the mannose-binding pockets of FimH variants 2, 5, 9, and 26 had identical amino acid sequences (amino acid positions in the lectin domains 1, 13, 44 to 54, and 132 to 142) (48). In addition to amino acids in the binding pocket, amino acids near the binding pocket can also influence binding due to their different properties in hydrophilicity, acidity, or basicity and steric hindrance (49). In comparison to FimH variant 2, the affinity of FimH variant 5 to GP2 was increased. Variant 5 has an amino acid exchange (A202V) in the pilin domain that could have increased the binding. Also, another alanine-to-valine exchange (A119V) in the lectin domain in FimH variant 9 resulted in higher binding to GP2 than FimH variant 2. This result showed that amino acid exchanges in both domains, namely, the lectin and pilin domains, affected the binding to GP2. Iebba et al. (43) found associations between certain *FimH* mutations in E. coli strains and inflammatory bowel diseases. In particular, mutations V27A and G66S in the lectin domain of FimH were related to Crohn’s disease. The same mutations occurred in 27 (V27A) and 3 FimH (G66S) variants in our study. The authors could also show that the A119V mutation which occurred in FimH variant 5 of this study was associated with adhesive E. coli. These E. coli bacteria were defined by Iebba et al. (43) as adhesive in a static adhesion assay with Caco-2 cells.

Aprikian et al. (50) showed that under flow conditions (more similar to the flow of contents in the intestine) FimH-receptor interactions can differ significantly from those measured under stationary conditions. Therefore, we selected two FimH variants and performed SPR experiments with all six GP2 isoforms. SPR experiments confirmed the ELISA results of interactions between each FimH variant and bovine GP2 and FimH variant 5 and porcine GP2. However, unlike in ELISA, FimH variant 9 bound to both porcine GP2 isoforms similarly in SPR, and there was no difference between the short and the long GP2 isoforms. Differences in binding of FimH to its receptors under static and shear stress conditions were reported elsewhere (51). In some cases, shear stress enhanced the strength of FimH binding, changing the low-binding phenotype into a high-binding phenotype (52). Le Trong et al. (53) showed that the inactive low-affinity state, triggered by an interaction between the lectin and pilin domains, can be lifted by a tensile force. We confirmed that GP2 from different species and different GP2 isoforms of one species interact differently with FimH under static and flow conditions. We therefore assume that the natural FimH-GP2 interaction in the gut depends on species-specific or even individual GP2 expression and its recognition by respective bacterial strains.

Studying more complex systems, such as natural and GP2-expressing bovine, human (both in this study), and porcine (18) intestinal cell lines, E. coli isolates showed similar binding activities to cells as to GP2 in the GP2 assay, e.g., isolates that bound well to GP2 also adhered well to the cell lines. The majority of isolates bound to cell lines with various bacterial numbers. Thus, E. coli isolates displayed a broad spectrum of binding activities and bound preferentially to porcine cells but less to bovine cells. Therefore, bovine cells appeared to be less susceptible to ETEC and EPEC infections than human and porcine cells. The binding activity of E. coli isolates to cell lines was neither pathotype dependent nor dependent on the host species of origin. Thus, cell culture models with only one cell type such as epithelial cells do not seem to be sufficient to clarify host specificity of E. coli, or indeed, most E. coli isolates do not exhibit such presumed host specificity and interspecies transfer is easy.

In general, the expression of GP2 in these cell lines did not affect adhesion, as the E. coli isolates adhered to them equally well or poorly. We confirmed our previous data studying adhesion/infection of Salmonella sp. to GP2-expressing cells (18). We can also conclude for E. coli that epithelial cell surfaces of cell lines seem to be saturated with glycoproteins serving as receptors for E. coli and Salmonella sp. It was shown that FimH-positive bacteria bind to GP2 on M cells. M cells might behave differently as they contain short or no microvilli and have a very thin glycocalix (37, 54) in contrast to intestinal epithelial cells which rather possess numerous microvilli surrounded by a thick glycocalix (55). However, we hypothesize that changes of the glycoprotein surface of intestinal epithelial cells during disease affect bacterial adhesion and consequently the intestinal microbiota. This assumption needs to be proven in further studies.

In this context, it was already shown that a high concentration of mucosal microbes, especially adherent bacteria, including adherent invasive E. coli, was found in patients with Crohn’s disease (CD) (5, 56). Interestingly, GP2 is also overexpressed in CD intestinal tissue and might be a link between the dysbiosis of the intestinal microbiota and inflammation.

GP2 is synthesized in the exocrine pancreas and transported into the intestinal lumen (57). We assume that, here, GP2 binds to bacteria and interferes with bacterial adhesion/infection to the intestinal wall and thus controls bacterial infection. To test this hypothesis, we preincubated ETEC and EPEC with soluble GP2 and infected wild-type and GP2-expressing bovine cells. ETEC adhesion to both cell types was inhibited by GP2, whereas adhesion of EPEC was not affected. Thus, pancreatic GP2 could control intestinal infections independently of the surface glycocalix composition of intestinal epithelial cells. This mechanism would open new possibilities for the prevention of ETEC infection. The fact that EPEC infection was not affected can be explained by differences in adhesion mechanisms. ETEC infections might depend more on initial FimH-receptor interactions, and it was shown that the enhanced production of highly mannosylated proteins on intestinal epithelia promoted FimH-mediated ETEC adhesion, while conversely, interruption of FimH lectin-epithelial interactions with soluble mannose, anti-FimH antibodies, or mutagenesis of *fimH* effectively blocked ETEC adhesion (23). In contrast, EPEC also uses adhesion strategies via bundle-forming pili (BFP) and independently from fimbriae via EspA, TIR, and intimin (58).

Independent of studies on GP2, it is known that the pancreas is connected with the digestive tract via a close communication loop. Dysfunction of this communication can lead to diseases of the pancreas or the digestive tract (59, 60). Initial studies in the rat model have shown that pancreatic functions are regulated by the intestinal microbiota. Thus, antimicrobial peptides (cathelicidin-related peptides [CRAMP]), formed by acinar cells, are identified as one component of pancreatic juice that affects the microbiota. Knockout of the gene Orai1was followed by the reduced secretion of these antimicrobial peptides and subsequently by increased bacterial colonization in the intestine. In contrast to supplementation with digestive enzymes, supplementation with CRAMP protected rats against intestinal dysbiosis and increased their survival rates (60).

It was shown that GP2 acts as an immunomodulator of innate and adaptive immune responses by decreasing proliferation, apoptosis, and activation of T cells, modulating cytokine secretion and inducing T cell attraction by intestinal epithelial cells (3). Here, we described a novel function of free (equivalent to pancreas derived) GP2 as it stimulated the activity of phagocytes against E. coli bacteria. Preincubation of bacteria with soluble GP2 enhanced phagocytosis rates in both porcine and human macrophages. Pancreatic GP2 forms high-molecular-weight aggregates in the pancreatic juice (29), potentially creating new epitopes which might affect binding to bacteria. It is known that proteins other than antibodies and complements can act as opsonins and promote the phagocytosis of bacteria (61).

In conclusion, we identified EPEC- and ETEC-associated FimH variants. FimH is an appropriate binding partner for GP2 regardless of the origin of GP2 and the E. coli pathotype. The FimH variants exhibit different affinity to GP2, preferentially to long GP2 isoforms. Differences were also found between static (GP2 assay) and dynamic conditions (SPR analysis) indicating that FimH-GP2 interactions are affected by shearing forces. On intestinal epithelial cell lines, within a complex intact glycocalyx, GP2 plays a minor role in the binding of E. coli. Free (pancreatic) GP2 is able to decrease ETEC adhesion to intestinal epithelia and to enhance bacterial phagocytosis by macrophages. Further studies should focus on the occurrence and function of GP2 during intestinal diseases.

## MATERIALS AND METHODS

### Bacterial isolates.

Intestinal pathogenic E. coli isolates of EPEC and ETEC pathotypes from porcine, bovine, and human as well as commensal E. coli were taken from our laboratory stock cultures, originally isolated from diarrheal and healthy animals and humans in the years 2010 to 2016. Every porcine and bovine isolate was from another pig or cattle producer. Every human isolate was from another person and collected during routine diagnostics. Of each E. coli pathotype and host, 20 different isolates were included (total of 180 isolates). The presence of crucial virulence factors/genes was verified by PCR for EPEC (intimin/*eae* gene) and ETEC (enterotoxins/*estIa*, *estII*, and *eltB* genes). The E. coli strains used for cloning and expression of proteins are explained in the respective paragraphs.

### Detection and analysis of *fimH* genes.

The presence of the *fimH* gene in E. coli isolates was confirmed by PCR using heat lysates of overnight cultures as the template. The PCR conditions were as follows: 98°C for 30 s; followed by 40 cycles of 98°C for 5 s, 60°C for 15 s, and 72°C for 45 s; and final extension at 72°C for 10 min. The primer sequences are listed in Table 3. PCR products were checked on a 1% agarose gel, and the *fimH*-positive samples were Sanger sequenced with the same primers commercially by LGC Genomics (Berlin, Germany). The results were analyzed with the software Unipro UGENE (62) and converted into a ClustalW format (.aln). Amino acid sequences were aligned and compared with MUSCLE (63) integrated into Unipro UGENE.

**TABLE 3:** PCR primer and FISH probe

Designation	Sequence (5′–3′)^*a*^	Size (bp)	Description	Reference
Gene target for primer				
FimH	f: GTTACAGGTCAGAGCATTGAC r: CGTCTTATCTGGCCTACAAAG	1,099	Type 1 fimbrial adhesin	This study
GP2	f: CTGCGGAAACATTCTGGAGAG r: ATGTTCAGGGAACTTACGATGG	180	Glycoprotein 2 for qPCR	This study
RPLP0	f: AAATGTTTCATTGTGGGAGC r: ATATGAGGCAGCAGTTTCTC	170	Reference gene for qPCR	72
Eubacteria FISH probe	GCWGCCWCCCGTAGGWGT		EUB338 Atto647N probe	76

a f, forward; r, reverse.

### Recombinant expression and purification of bovine GP2 isoforms.

Small tissue samples were taken from the pancreas of a freshly killed cow and preserved in RNAlater solution (Qiagen, Hilden, Germany). Then, RNA was extracted using an RNeasy minikit (Qiagen), and the reverse transcription was performed with the Maxima first-strand synthesis kit for RT-qPCR (ThermoFisher Scientific, Waltham, USA). The GP2 sequences were amplified and cloned into the pJET1.2 plasmid (ThermoFisher Scientific) and were Sanger sequenced. In order to express the secreted form of GP2, the GPI anchor was determined using the PredGPI software (64) and not cloned in the next step. Two bovine GP2 isoforms were cloned via the Gateway system into pDONR221 and pDEST8 plasmids (ThermoFisher Scientific). The DH10Bac bacteria were used to generate recombinant bacmids. The following GP2 expression was performed according to Roggenbuck et al. (29) with minor modifications. After isolation of the recombinant bacmids, Sf9 insect cells were transfected and baculovirus was amplified. Subsequently, the baculovirus titer was determined with a BacPAK baculovirus rapid titer kit (Clontech Laboratories, Mountain View, USA). Then, the two bovine GP2 isoforms were expressed in large volumes of Sf9 insect cell suspension cultures (200 mL). Purification of His-tagged GP2 was performed by immobilized metal affinity chromatography, and the purity was checked by SDS-PAGE followed by silver staining (65). Additionally, deglycosylation of GP2 was performed under denaturing conditions with the protein deglycosylation mix (New England BioLabs, Frankfurt am Main, Germany). Finally, the samples were tested by Western blotting with anti-6×-His antibodies and concanavalin A (ConA) (66) as first antibodies and polyclonal anti-rabbit IgG horseradish peroxidase (HRP; Sigma-Aldrich, St. Louis, USA) and streptavidin-HRP as secondary antibodies. Both GP2-purified recombinant isoforms were sent to Pineda Antibody Service (Berlin, Germany) to immunize rabbits and generate antibodies. After 110 days, rabbits were bled to collect sera with total IgG fraction. Recombinant human and porcine GP2s were produced similarly, which was mentioned in Kolenda et al. (18).

### GP2 assay.

GP2 assays were performed in flat-bottom 96-well plates (Nunc MaxiSorp). The plates were coated with 2.5 μg/mL purified recombinant GP2, 5 μg/mL RNase B (Sigma-Aldrich), or 5 μg/mL BSA (Roth, Karlsruhe Germany) in 0.1 M Na_2_CO_3_ (pH 9.6). After overnight incubation at 4°C, the plates were washed once with 1% BSA in phosphate-buffered saline (PBS), dried at room temperature for 30 min, and used for further experiments. T1F expression of E. coli was stimulated by cultivation of bacteria under static conditions for 48 h at 37°C in 500 μL LB media (67). Bacterial suspensions with 5 × 10^6^ CFU/mL were prepared in 1% BSA in PBS, and 100 μL of this suspension was pipetted into each of the protein-coated wells. For treatment with d-mannose or d-glucose, the bacteria were first incubated for 1 h at room temperature in 0.2 M d-mannose or d-glucose. The bacterial adhesion was allowed at room temperature for 2 h, and the unbound bacteria were removed by three wash steps with PBS. The attached bacteria were fixed with 4% paraformaldehyde (PFA) for 1 h at 4°C. After being washed three times with PBS, the bacteria were stained with 20 μg/mL propidium iodide (PI) for 15 min at room temperature in the dark. The plates were washed once with PBS, and finally, 100-μL fluorescence-labeled PolyAn Blue PMMA beads (PolyAn, Berlin, Germany) were pipetted into each well as a focusing aid for our VideoScan technology (68). Experiments were performed five times in duplicates. Ten images per well were taken, and the bacteria were counted by the software MaxiSlider. This software was developed in our laboratory to detect and count bacteria by size and shape. Bacteria were calculated as the number of bacteria per mm^2^. The median of adherent bacteria was determined for each well.

### Expression of FimH.

Expression of FimH was quantified using 12 E. coli isolates of 2 different FimH variants. Culturing and staining were performed in 1.5-mL reaction vials. The bacteria were cultured in LB media for 48 h under static conditions, centrifuged, washed once with PBS, and fixed with 4% PFA for 1 h at 4°C. After bacteria were washed once with PBS, nonspecific binding of antibodies was blocked with 1% BSA in PBS for 30 min at room temperature. The bacteria were incubated with a primary anti-FimH antibody from Sokurenko (69) at room temperature for 1 h followed by incubation with a secondary anti-rabbit IgG Alexa Fluor 647-labeled antibody (Dianova, Hamburg, Germany) for 1 h at room temperature. Finally, the bacterial concentrations were adjusted to an optical density at 600 nm (OD_600_) of 2, and 50 μL of each suspension was added to each well of the 96-well plate. The fluorescence intensity was measured using the VideoScan technology. The assay was performed three times in triplicates.

### Binding studies between FimH and GP2.

Genes of four FimH variants (variants 2, 5, 9, and 26) were cloned into the BamHI and HindIII restriction sites of plasmid pJET1.2, and the digested products were ligated into plasmid pQE-12. In addition, an N-terminal overhang of FimG, which is necessary for a correct FimH structure, and a His-Tag were cloned into the Bsu15I and HindIII sites of the plasmid pQE-12_FimH. The *fimH* gene sequences were confirmed by Sanger sequencing by LGC Genomics. The plasmids pQE-12_dscFimH-His_6_ were transformed into T7 Express E. coli (New England BioLabs) for the expression of FimH variants. Extraction of FimH proteins from the periplasmic space was performed according to Slonim et al. (70) and QIAexpressionist (71). The FimH was purified by immobilized metal affinity chromatography, and the purity was checked by SDS-PAGE with Coomassie brilliant blue staining. The GP2-FimH interaction was characterized initially by enzyme-linked immunosorbent assays (ELISAs). The GP2 isoforms were coated on 96-well plates like in section “GP2 assay” and were incubated with 2.5 μg/mL FimH for 1 h. Unbound FimH was washed away four times with PBS. The bound FimH was detected with a rabbit anti-FimH serum from Sokurenko (69) and a secondary anti-rabbit IgG HRP-labeled antibody (Sigma-Aldrich). Between these two incubation steps, the plates were washed four times with PBS. After a last washing step with PBS, a 3,3′,5,5′-tetramethylbenzidine (TMB) solution (Seramun Diagnostica, Heidesee, Germany) was added into the wells, and the reaction was stopped after 10 min by adding 0.25 M sulfuric acid. The mean absorbance intensity of the wells was quantified using the Sunrise absorbance reader (Tecan, Männedorf, Switzerland) at a wavelength of 450 nm and a reference wavelength of 620 nm.

To characterize the GP2 and FimH interaction under flow conditions, surface plasmon resonance (SPR) was applied using a BIAcore T200 system (GE Healthcare, Chicago, USA). Four purified recombinant GP2 isoforms, namely, GP2#Ho2, GP2#Ho4, GP2#Su1, and GP2#Bo1 were immobilized on CM5 sensor chips using an amine coupling kit (GE Healthcare) according to the manufacturer’s instructions. The GP2 isoforms were diluted in 10 mM acetate (pH 4.5) and immobilized at a level of 2,000 response units (RU). All experiments were performed at 25°C with a flow rate of 30 μL/min. First, the chips were equilibrated with 1× PBS-*P*+ buffer (GE Healthcare). Next, purified recombinant FimH proteins were injected at the following different concentrations: 0.1, 0.5, 2.0, 5.0, and 10 μM. The association time was kept 3 min, followed by dissociation with 1× PBS-*P*+ buffer for 5 min. Finally, the GP2-coated sensor surface was regenerated with 50 mM NaOH for 20 sec. Three FimH variants with the best binding to GP2 were also immobilized on CM5 sensor chips (target amount, 2,000 RU) and used for analysis with all six GP2 isoforms (0.5, 2.0, 6.0, 16, and 29 μM). The association time was kept at 3 minutes, while the dissociation time was increased to 10 minutes.

### Generation of GP2-expressing bovine cell lines.

GP2-expressing bovine cell lines were generated with the Lenti-X lentiviral expression system (Clontech Laboratories). The two bovine GP2 sequences from section “Recombinant expression and purification of bovine GP2 isoforms” were cloned with a GPI anchor (integration of GP2 into the cytoplasma membrane) into BamHI and EcoRI sites of the plasmid pLVX-IRES-puro. An empty plasmid (without GP2 sequence) was used as a negative control. The lentiviruses were produced according to the manufacturer’s protocol (Lenti-X lentiviral expression systems; Clontech Laboratories). Prior to the transduction of the bovine cell line FBJ, the puromycin tolerance limit was determined by 3-(4,5-dimethyl-2-thiazolyl)-2,5-diphenyl-2H-tetrazolium bromide (MTT) test in which the cell viability and proliferation were measured. The final transduced cell lines each contained one of the two GP2 isoforms or the empty vector. The successful expression of GP2 was confirmed by qPCR. The RNA of cell lines was extracted with the RNeasy minikit (Qiagen). Reverse transcription was done with the RevertAid H minus first strand cDNA synthesis kit (ThermoFisher Scientific). Finally, the qPCR was performed with a CFX96 touch real-time PCR detection system (Bio-Rad, Hercules, USA) and the following conditions: 95°C for 3 min; and then 40 cycles of 95°C for 30 s, 57°C for 30 s, and 72°C for 1 min. Additionally, *RPLP0* was used as a reference gene for the quantification of GP2 mRNA (72). The primer sequences are shown in Table 3. Indirect immunofluorescence (IIF) according to Lewis Carl et al. (73) with minor modifications was used as a second method to confirm GP2 expression. The cell lines were incubated with rabbit anti-GP2 sera, and the anti-rabbit IgG Alexa Fluor 647 (Dianova) was used as secondary antibody to confirm the expression on the cell line surface using VideoScan technology. The GP2-expressing IPEC-J2 cells were taken from Kolenda et al. (18), whereas the human LoVo cells expressing human GP2 were generated in parallel to the bovine FBJ.

### Adhesion assay.

Adhesion assays were performed with 90 E. coli isolates (10 isolates of each host species/pathotype) according to Kolenda et al. (18) and Frömmel et al. (74). The GP2-expressing bovine FBJ cells (this study), porcine IPEC-J2 cells (18), and human LoVo cells (this study) were cultured in flat-bottom 96-well plates (Nunc, MaxiSorp) at 37°C and 5% CO_2_ to confluence. The cell monolayers were washed twice with PBS and incubated in Dulbecco’s modified Eagle’s medium (DMEM)/Ham’s F-12 medium (Merck, Darmstadt, Germany) supplemented with 5% FBS and 2 mM l-glutamine. The bacteria were cultivated under static conditions for 48 h at 37°C, 5 × 10^6^ CFU/mL was prepared in cell culture media, and 50 μL was added to each well of the plate. For additional GP2 incubation, the bacteria were preincubated with 10 μg/mL GP2 in cell culture media for 1 h at room temperature. The plates were incubated for 2 h at 37°C and 5% CO_2_ and washed three times with PBS, and fixation was done with 4% PFA for 1 h at 4°C. The plates were washed three times with PBS before 95% ethanol was added. The ethanol was removed after 5 min, and the plates were air dried. The bacteria were stained by fluorescence *in situ* hybridization (FISH) with the EUB338 Atto647N probe (biomers.net, Ulm, Germany) which recognizes all bacteria (75, 76). The probe was diluted in freshly prepared hybridization buffer (0.9 M NaCl, 20 mM Tris-HCl, 0.01% SDS, and 15% formamide) to a final concentration of 5 ng/μL. Subsequently, 40 μL was dispensed to each well, and the plates were incubated for 1 h at 46°C in a humid chamber. After a single washing step with prewarmed washing buffer (0.9 M NaCl, 20 mM Tris-HCl, and 0.01% SDS), the plates were incubated for 10 min at 48°C in the same buffer. The cell lines were washed once with PBS and cell nuclei were stained with 50 μg/mL 4′,6-diamidino-2-phenylindole (DAPI). Finally, the plates were stored with PBS at 4°C in the dark until measured with the VideoScan technology. All experiments were performed five times in duplicates.

### Phagocytosis assay.

Phagocytosis assays were performed with human (THP-1) and porcine (3D4/31) macrophage cell lines. Cell lines were cultured in 96-well plates until 90% to 95% confluence. THP-1 cells were induced with 0.05 μg/mL phorbol 12-myristate 13-acetate (PMA), and after 24 h of rest, cells were ready to use. For 3D4/31 cells, there was no need for induction because of the active form of this cell line. On assay day, bacterial strains (EPEC) were incubated with GP2 (10 μg/mL) for 1 h. Negative-control bacteria were incubated with PBS. Then, 10^5^ bacteria per well were incubated on macrophages for 30 min at 37°C, and after subsequent washing with PBS, wells were rinsed with 0.5× cold trypsin-EDTA. Cells were fixed with 4% PFA for 1 h, permeabilized with 95% ethanol for 5 min, and washed again with PBS; bacteria were stained with FISH using the EUB338 Atto647N probe as described in the section “Adhesion assay.” THP-1 cells were stained with phalloidin, whereas 3D4/31 cells were not stained due to self-fluorescence. Finally, cell nuclei were stained with DAPI and plates were measured with VideoScan technology. Experiments were performed three times in triplicates.

### VideoScan technology.

The VideoScan technology is based on fluorescence microscopy (68, 77) where bacteria and eukaryotic cells are stained with dyes that emit at different wavelengths. This technology makes it possible to determine the fluorescence intensities of fluorescent objects and calculate their object areas which results in the recognition and enumeration of eukaryotic cells and bacteria. The microscope IX83 (Olympus, Shinjuku, Japan) works with a ×20 magnification objective. First, the system needs a plane to focus (DAPI-stained nuclei or PolyAn blue polymethylmethacrylate [PMMA] focus beads). Then, images are taken by the camera acA1920-40 μm (Basler, Ahrensburg, Germany) in the DAPI, Cy5 (FISH), and Cy3 (PI) channels. In the current study, a total of 10 images per well were taken, and bacteria were counted. Experiments for each recombinant protein and the cell line were performed in duplicates five times.

### Statistical analysis.

All results were evaluated statistically and graphically with the R software version 3.6.1 (78). RStudio version 1.2.1335 was the user interface for writing the R scripts. Medians and median absolute deviations were determined for all measurements. The modified pairwise Mood’s median test according to Fligner and Rust (79) with a *P* value limit of 5% was used for the significance analyses of the assays. All graphics were designed with the R integrated package ggplot2 (80).

### Data availability.

The E. coli isolates used in this study can be made available to researchers by contacting the corresponding author.
