# Supplementary material for: Adhesion of Enteropathogenic, Enterotoxigenic, and Commensal Escherichia coli to the Major Zymogen Granule Membrane Glycoprotein 2
Source: Appl Environ Microbiol. 2022 Mar 8;88(5):e02279-21. doi: 10.1128/aem.02279-21 (PMC8904060; doi:10.1128/aem.02279-21)
Supplement: Supplemental file 1 — Fig. S1 to S4 and Table S1. Download aem.02279-21-s0001.pdf, PDF file, 0.8 MB [file aem.02279-21-s0001.pdf]

Adhesion of enteropathogenic, enterotoxigenic and commensal *Escherichia coli* to the Major Zymogen Granule Membrane Glycoprotein 2

Christin Bartlitz[a], Rafał Kolenda[b], Jarosław Chilimoniuk[c], Krzysztof Grzymajło[b], Stefan Rödiger[a,h], Rolf Bauerfeind[d], Aamir Ali[e,f], Veronika Tchesnokova[g], Dirk Roggenbuck[a,h], Peter Schierack[a,h]#

[a]Institute of Biotechnology, Faculty Environment and Natural Sciences, Brandenburg University of Technology Cottbus-Senftenberg, Senftenberg, Germany

[b]Department of Biochemistry and Molecular Biology, Faculty of Veterinary Medicine, Wrocław University of Environmental and Life Sciences, Wrocław, Poland

[c]Department of Genomics, Faculty of Biotechnology, University of Wrocław, Wrocław, Poland

[d]Institute for Hygiene and Infectious Diseases of Animals, Justus Liebig University, Giessen, Germany

[e]National Institute for Biotechnology and Genetic Engineering (NIBGE), Faisalabad, Pakistan

[f]Pakistan Institute of Engineering and Applied Sciences (PIEAS), Islamabad, Pakistan

[g]Department of Microbiology, University of Washington, Seattle, Washington, USA

[h] Faculty of Health Sciences Brandenburg, Brandenburg University of Technology Cottbus-Senftenberg, Senftenberg, Germany

Running Head: Adhesion of *E. coli* to Glycoprotein 2

#Address correspondence to Peter Schierack, Institute of Biotechnology, Faculty Environment and Natural Sciences, Brandenburg University of Technology Cottbus-Senftenberg, Universitätsplatz 1, 01968 Senftenberg; peter.schierack@b-tu.de.

Key words

major zymogen granule membrane glycoprotein 2 (GP2), *Escherichia coli*, FimH, receptor, adhesion, intestinal cell lines

SUPPLEMENTARY MATERIAL

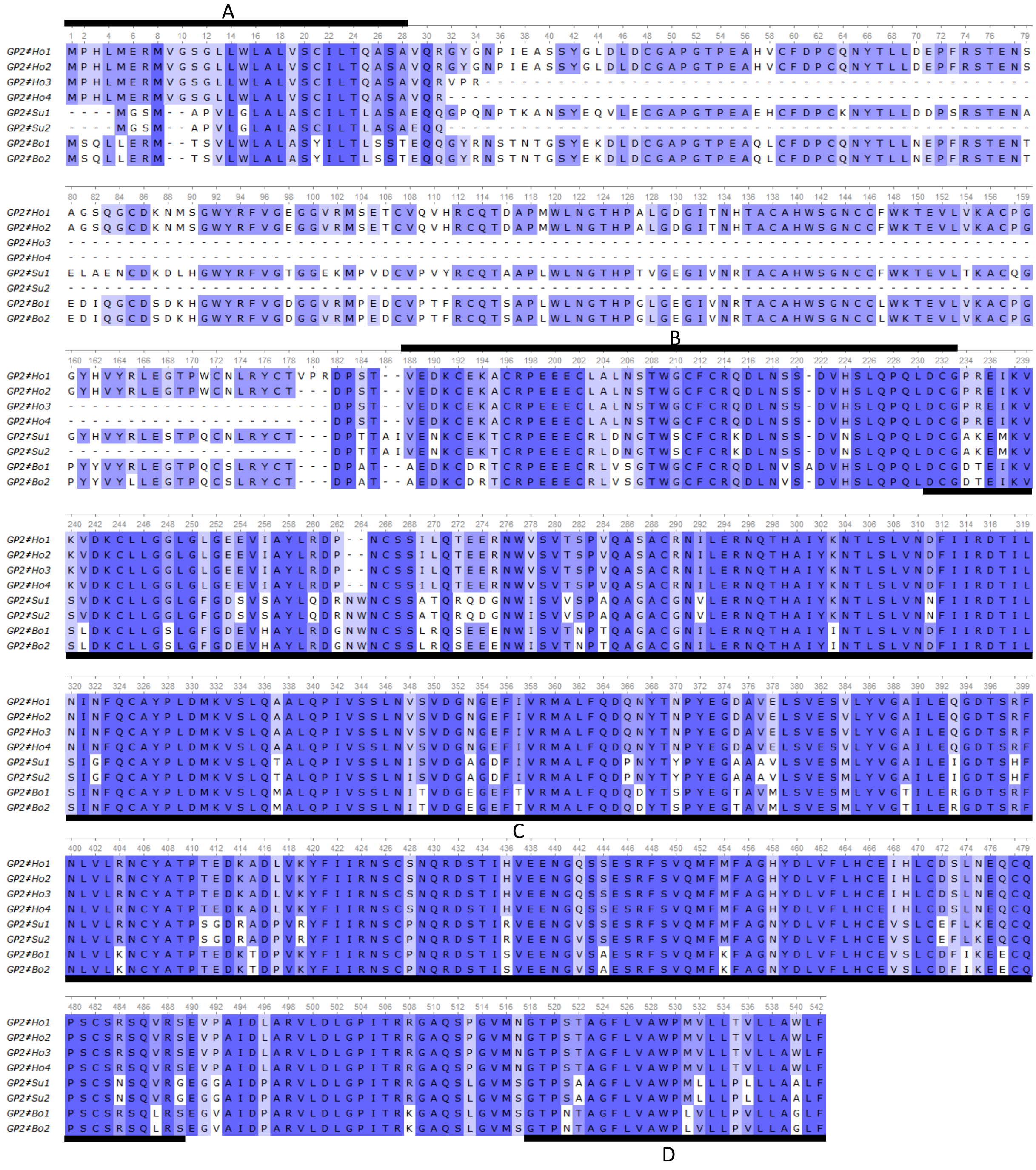

Figure S1. GP2 amino acid sequences from humans, pigs and cattle. The human and porcine sequences can be found in the GenBank under the following numbers GP2#Ho1: NP001007241, GP2#Ho2: NP001493, GP2#Ho3: NP001007242, GP2#Ho4: NP001007243, GP2#Su1: KU665995 and GP2#Su2: KU665994. Particularly high matches occur in the

marked areas. A: N-terminal signal peptide, B: EGF-like domain, C: ZP domain, D: C-terminal GPI anchor.

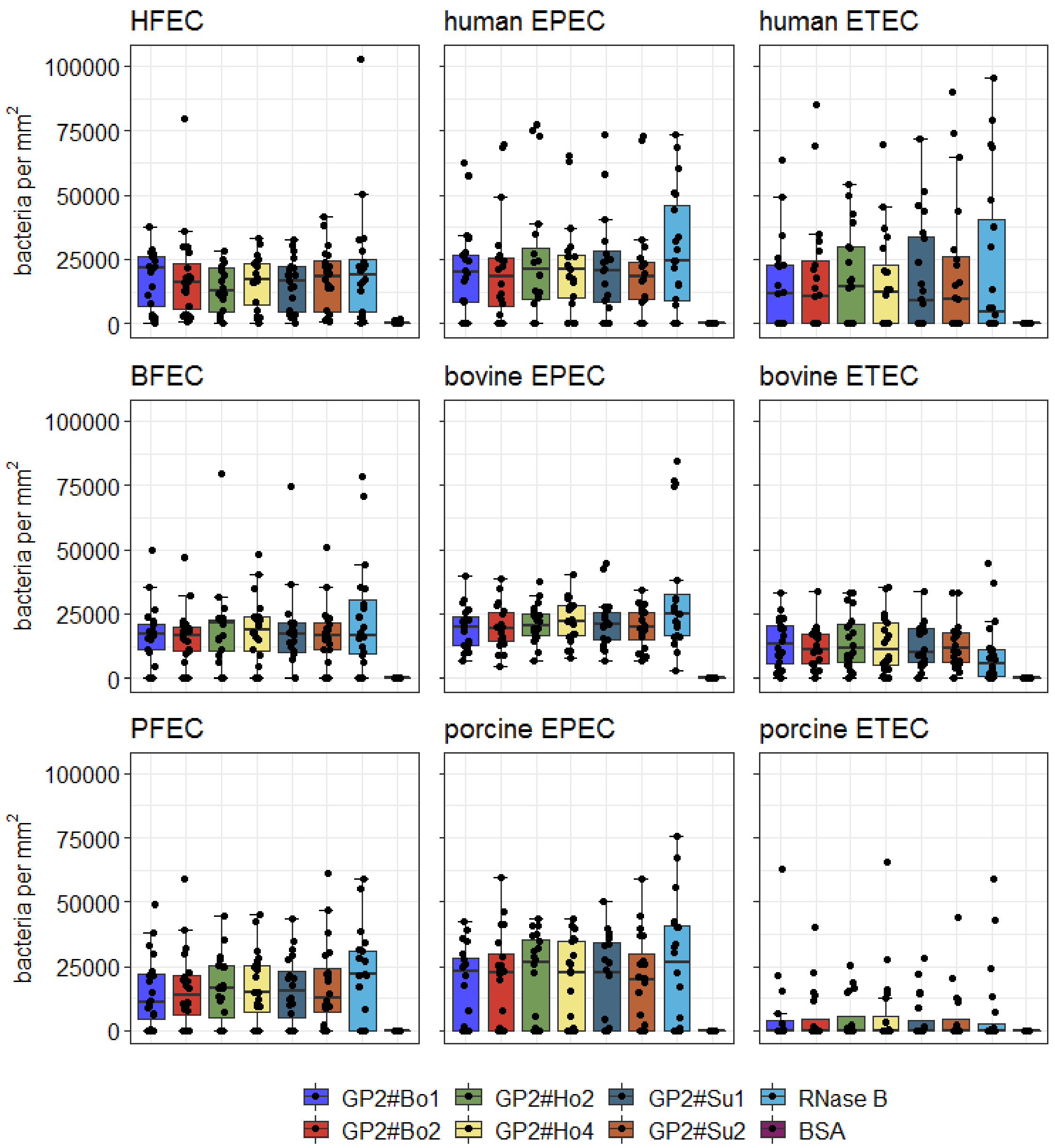

Figure S2. Binding of different *E. coli* pathotypes of human, bovine, and porcine origin to six GP2 isoforms, RNase B and BSA (n=20). RNase B was used as standard positive control glycoprotein, while BSA was used as negative control protein. The experiment was performed three times in duplicates. GP2#Bo1: bovine GP2 isoform 1, GP2#Bo2: bovine GP2 isoform 2,

GP2#Ho2: human GP2 isoform 2, GP2#Ho4: human GP2 isoform 4, GP2#Su1: porcine GP2 isoform 1, GP2#Su2: porcine GP2 isoform 2.

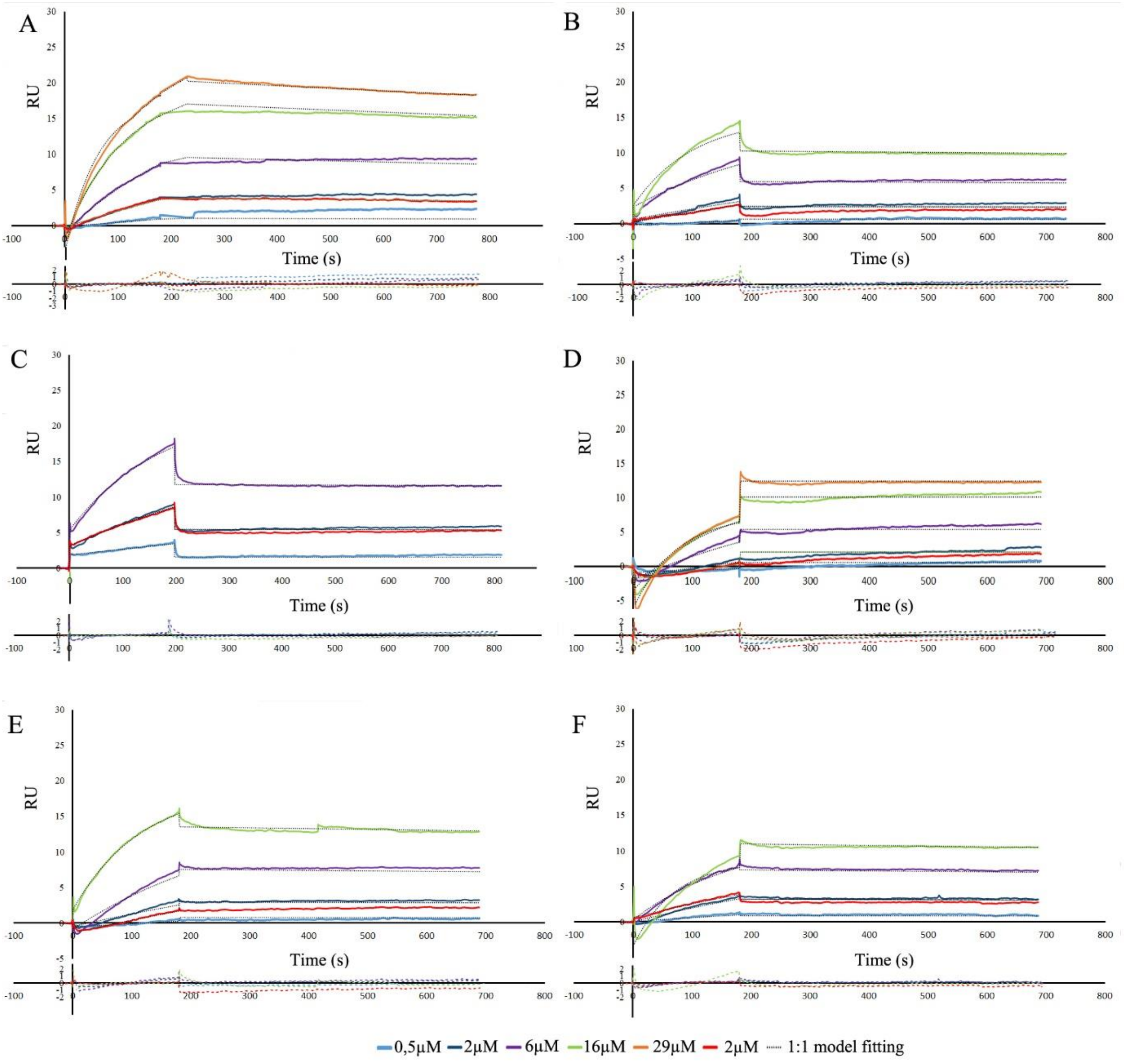

Figure S3. Interaction of various GP2 isoforms (A: GP2#Bo1; B: GP2#Bo2; C: GP2#Ho2; D: GP2#Ho4; E: GP2#Su1; F: GP2#Su2) with immobilized FimH variant 9 analysed with an SPR. Lines represent different concentrations of the analyte (0.5, 2.0, 6.0, 16, 29 µM) in PBS-P+ buffer. Binding data were collected at a flow rate of 30 µl/minutes. Below each sensorgram residual plots are presented, defining differences between experimental data and fitted kinetic model.

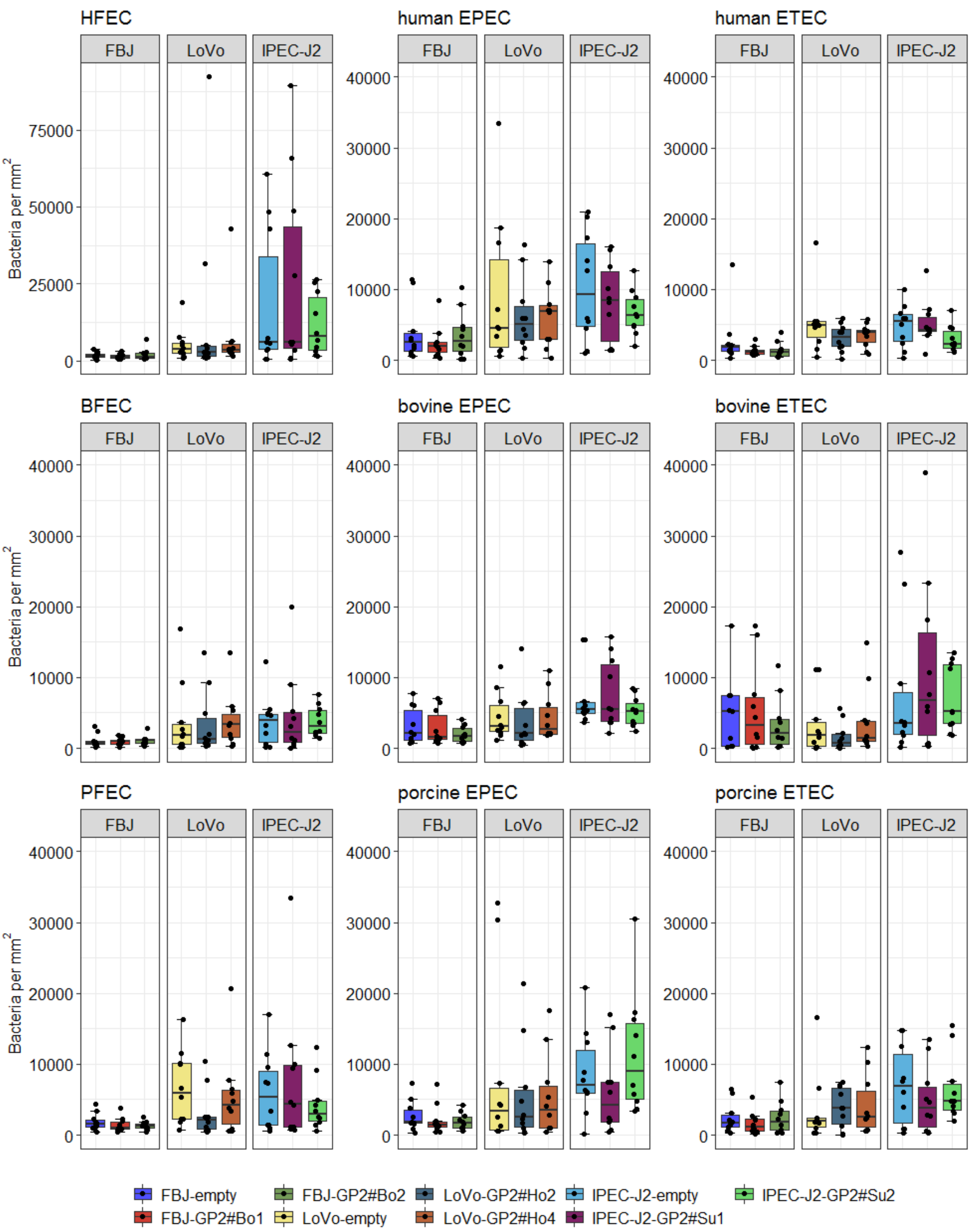

Figure S4. Adhesion of *E. coli* from different pathogeny and origin to cell lines (n = 10). HFEC: human commensal *E. coli*, BFEC: bovine commensal *E. coli*, PFEC: porcine commensal *E. coli*, FBJ/LoVo/IPEC-J2-empty: no GP2 expression on FBJ/LoVo/IPEC-J2 cell lines, FBJ-GP2#Bo1/Bo2: bovine GP2 isoform 1/2 expressed on FBJ cell line, LoVo-

GP2#Ho2/Ho4: human GP2 isoform 2/4 expressed on LoVo cell line, IPEC-J2-

GP2#Su1/Su2: porcine GP2 isoform ½ expressed on IPEC-J2 cell line.

Table S1. *E. coli* isolate name (number), pathotype, host origin, FimH variant type, and binding capability to six different GP2 isoforms

- … no binding, o … low binding, + … medium binding, ++ … high binding

Number	Pathotype	Host origin	FimH variant	Binding to GP2 isoforms					
				GP2 #Bo1	GP2 #Bo2	GP2 #Ho2	GP2 #Ho4	GP2 #Su1	GP2 #Su2
91	commensal	porcine	1	+	+	+	+	+	+
5547	commensal	human	1	o	o	o	o	o	o
5682	ETEC	human	1	-	-	-	-	-	-
5689	ETEC	human	1	-	-	-	-	-	-
5690	ETEC	human	1	-	-	-	-	-	-
5691	ETEC	human	1	-	-	-	-	-	-
5998	EPEC	porcine	1	o	-	-	o	-	o
6586	commensal	bovine	1	+	+	+	+	+	+
6595	commensal	bovine	1	-	-	-	-	-	-
7501	EPEC	bovine	1	+	+	+	+	+	+
7503	EPEC	bovine	1	+	+	+	+	+	+
7506	EPEC	bovine	1	+	+	+	+	+	+
7507	EPEC	bovine	1	+	+	+	+	+	+
7509	EPEC	bovine	1	+	+	+	+	+	+
7514	EPEC	bovine	1	+	+	+	+	+	+
7516	EPEC	bovine	1	o	o	+	+	+	o
7528	EPEC	bovine	1	+	+	+	+	+	o
7551	ETEC	Porcine	1	-	-	-	-	-	-
122	commensal	porcine	2	-	-	-	-	-	-
136	commensal	porcine	2	+	+	+	+	+	+
4297	commensal	porcine	2	+	+	+	+	+	+
4318	commensal	porcine	2	+	+	+	+	+	+
4320	commensal	porcine	2	+	+	+	+	+	+
4401	commensal	porcine	2	+	+	+	++	+	++
4445	commensal	porcine	2	+	+	+	+	+	o
4732	commensal	porcine	2	+	+	+	+	+	+

5200	ETEC	human	2	+	+	+	+	++	+
5201	ETEC	human	2	+	+	++	+	++	++
5204	ETEC	human	2	+	+	+	+	++	++
5205	ETEC	human	2	+	++	+	++	+	++
5263	EPEC	human	2	++	++	++	++	++	++
5268	EPEC	human	2	++	++	++	++	++	++
5281	EPEC	human	2	o	o	o	+	o	+
5282	EPEC	human	2	+	o	+	+	o	o
5285	EPEC	human	2	o	o	+	+	o	+
5289	EPEC	human	2	+	+	+	+	+	+
5527	commensal	human	2	+	+	+	+	+	+
5529	commensal	human	2	+	+	+	+	+	+
5536	commensal	human	2	+	+	+	+	+	+
5543	commensal	human	2	+	+	+	+	+	+
5631	EPEC	human	2	+	+	+	+	+	+
5637	ETEC	human	2	++	++	++	++	++	++
5638	ETEC	human	2	-	-	-	-	-	-
5652	EPEC	human	2	-	-	-	-	-	-
5655	EPEC	human	2	-	-	-	-	-	-
5680	ETEC	human	2	++	+	++	+	+	+
5692	ETEC	human	2	-	-	-	-	-	-
5981	EPEC	porcine	2	+	++	+	+	++	++
5982	EPEC	porcine	2	+	+	++	+	+	+
5983	EPEC	porcine	2	+	+	+	+	+	+
5984	EPEC	porcine	2	+	++	+	+	+	++
5985	EPEC	porcine	2	o	o	o	o	o	o
5988	EPEC	porcine	2	+	+	++	++	+	+
5989	EPEC	porcine	2	-	-	-	-	-	-
5990	EPEC	porcine	2	+	+	+	+	+	+
5991	EPEC	porcine	2	+	+	+	+	+	+
5992	EPEC	porcine	2	-	-	-	-	-	-
5996	EPEC	porcine	2	-	-	-	-	-	-
5997	EPEC	porcine	2	+	+	+	+	+	+
5999	EPEC	porcine	2	+	+	+	+	+	+
6573	commensal	bovine	2	o	o	o	o	o	o
6576	commensal	bovine	2	++	++	++	++	++	++
6583	commensal	bovine	2	+	+	o	+	+	+
6587	commensal	bovine	2	+	+	+	++	+	+
6588	commensal	bovine	2	+	+	+	+	+	+

6589	commensal	bovine	2	+	+	+	+	+	+
6590	commensal	bovine	2	+	+	+	+	+	+
6592	commensal	bovine	2	+	+	+	+	+	+
6593	commensal	bovine	2	+	+	+	+	+	+
6594	commensal	bovine	2	+	+	+	+	+	+
6597	commensal	bovine	2	+	+	+	+	+	+
6602	commensal	bovine	2	+	+	+	+	+	+
6604	commensal	bovine	2	+	+	+	+	+	+
7500	EPEC	bovine	2	+	+	+	+	++	+
7505	EPEC	bovine	2	+	+	+	+	+	+
7510	EPEC	bovine	2	+	+	+	++	++	+
7524	EPEC	bovine	2	+	+	+	+	+	+
7527	EPEC	bovine	2	+	o	+	+	+	o
7533	EPEC	bovine	2	+	+	+	+	+	+
7546	ETEC	porcine	2	++	+	+	++	+	+
7549	ETEC	porcine	2	-	-	-	-	-	-
7550	ETEC	porcine	2	o	+	+	+	o	+
7554	ETEC	porcine	2	+	+	+	+	+	+
148	commensal	porcine	3	o	o	+	+	o	o
4298	commensal	porcine	3	o	+	+	+	+	+
5206	ETEC	human	3	+	+	+	+	o	+
5288	ETEC	human	3	+	+	+	+	+	+
5532	commensal	human	3	o	o	o	o	o	o
4136	commensal	porcine	4	+	+	+	+	+	+
4374	commensal	porcine	4	++	++	++	++	++	++
5211	EPEC	human	4	o	+	+	o	+	o
5267	EPEC	human	4	+	+	+	+	+	+
5993	EPEC	porcine	4	-	-	-	-	-	-
6584	commensal	bovine	4	-	-	-	-	-	-
7535	ETEC	porcine	4	-	-	o	-	o	-
7553	ETEC	porcine	4	+	+	+	+	+	+
4305	commensal	porcine	5	-	-	-	-	-	-
5202	ETEC	human	5	-	-	-	-	-	-
4310	commensal	porcine	6	-	-	-	-	-	o
4418	commensal	porcine	6	+	+	+	+	+	+
5980	EPEC	porcine	6	-	-	-	-	-	-
6596	commensal	bovine	6	+	+	+	+	+	+
7552	ETEC	porcine	6	o	o	o	o	o	o
5207	EPEC	human	7	+	++	++	+	++	+

5283	EPEC	human	7	-	-	-	-	-	-
5284	EPEC	human	7	-	-	-	-	-	-
5995	EPEC	porcine	7	+	+	+	+	+	+
7519	EPEC	bovine	7	+	+	+	+	+	+
5208	EPEC	human	8	+	+	+	+	+	+
5209	EPEC	human	8	+	+	+	+	++	+
5540	commensal	human	8	+	+	o	+	o	+
5542	commensal	human	8	o	o	o	o	o	o
5545	commensal	human	8	+	+	+	+	+	+
5549	commensal	human	8	+	+	+	+	+	+
5553	commensal	human	8	+	+	+	+	+	+
5554	commensal	human	8	+	+	+	+	+	+
5651	EPEC	human	8	+	+	+	+	+	+
5654	EPEC	human	8	+	+	+	+	+	+
5287	ETEC	human	9	+	+	+	+	+	+
5695	ETEC	human	9	+	+	+	+	+	o
5696	ETEC	human	9	+	+	+	+	o	o
7496	ETEC	bovine	9	o	+	o	o	o	o
7504	ETEC	bovine	9	o	o	o	-	o	o
7508	ETEC	bovine	9	+	+	+	+	+	+
7511	ETEC	bovine	9	o	+	o	o	o	+
7512	ETEC	bovine	9	-	-	-	-	-	-
7515	ETEC	bovine	9	o	o	o	o	o	o
7517	ETEC	bovine	9	+	+	+	+	+	+
7518	ETEC	bovine	9	o	o	o	-	+	o
7520	ETEC	bovine	9	+	+	o	+	+	+
7521	ETEC	bovine	9	+	o	o	o	o	+
7522	ETEC	bovine	9	+	+	+	+	+	+
7523	ETEC	bovine	9	o	o	o	o	o	o
7525	ETEC	bovine	9	+	+	+	+	o	+
7526	ETEC	bovine	9	o	o	+	o	o	o
7529	ETEC	bovine	9	+	+	+	+	+	o
7534	ETEC	bovine	9	+	+	+	+	+	+
7543	ETEC	porcine	9	-	-	-	-	-	-
5538	commensal	human	10	o	o	o	o	o	o
5986	EPEC	porcine	11	+	++	++	+	+	+
5987	EPEC	porcine	11	++	++	+	++	+	+
6580	commensal	bovine	12	+	o	+	o	o	+
6598	commensal	bovine	12	-	-	-	-	-	-

7498	ETEC	bovine	13	+	+	+	+	+	+	
7502	ETEC	bovine	13	o	o	+	o	o	o	
7530	ETEC	bovine	13	+	+	+	+	+	+	
7531	ETEC	bovine	13	+	+	+	+	+	+	
7499	EPEC	bovine	14	+	+	+	+	+	+	
7513	EPEC	bovine	14	o	o	o	o	o	o	
7532	EPEC	bovine	14	+	+	+	+	+	+	
7555	EPEC	bovine	15	+	+	+	+	+	+	
5539	commensal	human	16	+	+	+	+	+	+	
7536	ETEC	porcine	17	+	++	+	+	+	++	
7497	EPEC	bovine	18	+	+	+	+	+	+	
5541	commensal	human	19	+	+	+	+	+	+	
5537	commensal	human	20	+	+	+	+	+	+	
5531	commensal	human	21	+	++	+	+	+	++	
5212	EPEC	human	22	+	+	+	+	+	+	
5210	EPEC	human	23	+	+	+	+	+	+	
4781	commensal	porcine	24	-	-	-	-	-	-	
6605	commensal	bovine	25	+	+	+	+	+	+	
5994	EPEC	porcine	26	-	-	-	-	-	-	
5548	commensal	human	27	+	+	+	+	+	+	
5528	commensal	human	28	-	-	-	-	-	-	
4315	commensal	porcine	29	o	+	+	o	+	+	
4127	commensal	porcine	30	-	-	-	-	-	-	
4444	commensal	porcine	-	o	o	o	o	o	o	
5203	ETEC	human	-	-	-	-	-	-	-	
5551	commensal	human	-	o	o	o	o	o	o	
5686	ETEC	human	-	-	-	-	-	-	-	
7537	ETEC	porcine	-	-	-	-	-	-	-	
7538	ETEC	porcine	-	-	-	-	-	-	-	
7539	ETEC	porcine	-	-	-	-	-	-	-	
7540	ETEC	porcine	-	-	-	-	-	-	-	
7541	ETEC	porcine	-	-	-	-	-	-	-	
7542	ETEC	porcine	-	-	-	-	-	-	-	
7544	ETEC	porcine	-	-	-	-	-	-	-	
7545	ETEC	porcine	-	-	-	-	-	-	-	
7547	ETEC	porcine	-	-	-	-	-	-	-	
7548	ETEC	porcine	-	-	-	-	-	-	-	

Table S2. Relationship between FimH Variant and *fimH* alleles. FimH Variant number was assigned in the course of this study based on amino acid substituions in FimH precursor (full mature FimH with leader peptide). A unique FimH Variant can be product of multiple *fimH* alleles, with the latter's nomeclature derived from CH typer (https://cge.cbs.dtu.dk/services/chtyper/) and Enterobase Database (http://enterobase.warwick.ac.uk/species/ecoli) and used for prediction of a sequence type (ST, see Table S2).

**FimH Variant**	***fimH* allele(s)**
**1**	54
**2**	25, 31, 32, 35, 38, 61, 65, 82, 86, 87, 97, 289, 305, 331, 1566, 1569, 1570
**3**	27, 1567
**4**	24
**5**	23
**6**	54
**7**	90, 677
**8**	2, 9, 14, 88, 115
**9**	54
**10**	41
**11**	30
**12**	54
**13**	237
**14**	440
**15**	319
**16**	76
**17**	158
**18**	446
**19**	1568
**20**	22
**21**	56
**22**	57
**23**	12
**24**	1259
**25**	69
**26**	555
**29**	398
**30**	1183

Table S3. Association of *fimH* alleles found in this study with *E. coli* clonal groups and lineages. This association was determined by analyzing available MLST and CH typing data from multiple databases (unpublished data).

***fimH* allele**	**Most prevalent ST**	**Phylogroup**
**2**	ST127	B2
**9**	ST73	B2
**12**	ST73	B2
**14**	ST14	B2
**22**	ST131	B2
**23**	CC10 (multiple ST)	A
**24**	promiscuous: CC10 (ST43, ST34, ST752 etc.), CC88 (ST410 etc.), CC58 (ST443 etc.)	A, C, B1
**25**	promiscuous: CC58 (ST17 etc.), ST655	B1, C
**27**	promiscuous: CC10 (ST10 etc.), ST69, ST95, ST405, ST648	A, D, B2, D, F
**30**	ST131; less frequent: ST73 (like Nissle1917), ST95, CC10 (ST34 etc.)	B2, B2, B2, A
**31**	promiscous among B1 STs: CC58 (ST145, ST40), ST1196, ST453, ST278	B1
**32**	CC58 (ST58, ST155, ST55, ST162, ST442)	B1
**35**	ST23, ST448, ST75, ST359	C, B1, A, B1
**38**	ST158, ST297	B1
**41**	promiscuous: ST131, ST95, CC10 (ST10, ST48 etc.), ST59	B2, B2, A, F
**54**	CC10 (ST10, ST48 etc.)	A
**56**	ST405	D
**57**	CC1283 (ST15, ST1283)	B2
**61**	ST224	B1
**65**	ST38	D
**69**	ST216	A
**76**	ST998	B2
**82**	ST11	E
**86**	ST16	B1
**87**	ST196	B1
**88**	ST1092	B2
**90**	CC28 (ST28, ST121)	B2
**97**	ST117	F
**115**	CC625 (ST969, ST625)	B2
**158**	CC910 (ST910, ST2952)	C
**237**	promiscuous: ST2368, ST120 etc.	A, B1
**289**	promiscuous among B1 STs: ST86, ST711, ST5378 etc.	B1
**305**	ST607	A
**319**	ST337	B1
**331**	ST32	E
**398**	ST542	A
**400**	promiscuous among A STs: ST48, ST10, ST4247 etc.	A
**440**	ST21	B1

**446**	ST33	B1
**555**	ST206	B1
**677**	ST3053	B2
**1135**	data not available	.
**1183**	ST7875	unassigned
**1252**	ST5395	B1
**1259**	data not available	.
**1566**	data not available	.
**1567**	data not available	.
**1568**	data not available	.
**1569**	data not available	.
**1570**	data not available	.
